# Structural variation on the human Y chromosome from population-scale resequencing

**DOI:** 10.3325/cmj.2015.56.194

**Published:** 2015-06

**Authors:** Jose Rodrigo Flores Espinosa, Qasim Ayub, Yuan Chen, Yali Xue, Chris Tyler-Smith

**Affiliations:** 1 The Wellcome Trust Sanger Institute, Hinxton, UK; 2 Department of Ecology and Evolution, University of Lausanne, Lausanne, Switzerland

## Abstract

**Aim:**

To investigate the information about Y-structural variants (SVs) in the general population that could be obtained by low-coverage whole-genome sequencing.

**Methods:**

We investigated SVs on the male-specific portion of the Y chromosome in the 70 individuals from Africa, Europe, or East Asia sequenced as part of the 1000 Genomes Pilot project, using data from this project and from additional studies on the same samples. We applied a combination of read-depth and read-pair methods to discover candidate Y-SVs, followed by validation using information from the literature, independent sequence and single nucleotide polymorphism-chip data sets, and polymerase chain reaction experiments.

**Results:**

We validated 19 Y-SVs, 2 of which were novel. Non-reference allele counts ranged from 1 to 64. The regions richest in variation were the heterochromatic segments near the centromere or the *DYZ19* locus, followed by the ampliconic regions, but some Y-SVs were also present in the X-transposed and X-degenerate regions. In all, 5 of the 27 protein-coding gene families on the Y chromosome varied in copy number.

**Conclusions:**

We confirmed that Y-SVs were readily detected from low-coverage sequence data and were abundant on the chromosome. We also reported both common and rare Y-SVs that are novel.

Structural variation, here considered as genetic variation that affects more than a single base pair of DNA, accounts for the majority of the nucleotide differences between individuals (1). Furthermore, structural variants (SVs) have substantial functional impact. For example, individual SVs are more likely than individual single nucleotide polymorphisms (SNPs) to lead to phenotypic differences such as changes in gene expression (2), are responsible for more loss-of-function in protein-coding genes (3), and underlie many disorders, indeed leading to the recognition of a novel class of “genomic disorder” (4). There has therefore been considerable interest among human geneticists in cataloguing SVs in both patients and samples from the general population. Technological advances have allowed the resolution of genome surveys to increase from ~ 5 Mb in cytogenetic studies, via ~ 100 kb in comparative genomic hybridization (CGH) experiments based on bacterial artificial chromosome clones (5) to ~ 0.5 kb using high-resolution oligonucleotide arrays (6) and finally to base-pair resolution in sequence-based studies (7).

The Y chromosome (here, “Y chromosome” always refers to the male-specific portion excluding the pseudoautosomal regions) stands out in SV studies for two reasons. First, it is unusually rich in SVs (8). Cytogeneticists reported visible variation in the length of the Y long arm, pericentric inversions, and translocations of nucleolus organizer regions (NORs, containing ribosomal RNA gene clusters) to the Y in the general population (9). Early molecular studies focusing mainly on individual loci identified variable microsatellites (10), minisatellites (11,12), major satellites (13,14), tandemly-arranged genes (15), and also large duplications, deletions, and inversions (16,17) in the general population. The enrichment of copy number variants (CNVs) on the Y was confirmed by genome-wide surveys (5). More recently, however, the second reason for the Y standing out has come to the fore: it has been neglected in high-resolution studies, either because these are focused on women (6) or, in the most comprehensive sequence-based study (7), because of a combination of lower depth of coverage of the sequence reads (ie, less sequence information because of its haploid status), the use of SV discovery algorithms optimized for autosomes, and the complexities of mapping the short sequence reads produced by next-generation sequencing to the repeated regions that make up most of the Y.

There are, nevertheless, good reasons to study Y-SVs at high resolution. Several independent >100 kb deletions on Yq (18), and variation of the copy number of the *TSPY* array on Yp (19), contribute to spermatogenic variation and failure, but together account for only a small proportion of male infertility. Do smaller Y-SVs account for additional cases of male infertility? In forensic genetics, the *AMELY* locus forms the basis for the most commonly-used sex test, but is unreliable in a minority of men (20) because of deletions of this locus, which make men difficult to distinguish from women, and have several independent origins (21). More generally, the absence of recombination over most of the length of the Y provides an opportunity to investigate the density, size distribution, and mutational origins of SVs in this different environment, aided by a well-established phylogeny (22).

We therefore used a previously-generated population-scale resequencing data set to investigate Y-SVs at high resolution. We chose the 70 male samples used in the 1000 Genomes Pilot Project (23) because, despite their potential, only three validated Y-SVs have been reported in them (7), leaving scope for further discoveries, and additional high-coverage sequence data from 8 of these men have been released. We performed further Y-SV discovery in both the original low-coverage and newer high-coverage sequence data, combined these Y-SVs with those reported by Mills et al, Complete Genomics, and Phase 1 of the 1000 Genomes project in the same samples (7,24), and performed extensive validation and functional prediction on the combined set.

## Materials and methods

### 1000 Genomes data

We analyzed 70 Y chromosome sequences released by the 1000 Genomes Pilot 1 Project. These chromosomes were sequenced using ~ 36 bp Illumina (Illumina, Inc. San Diego, CA, USA) reads. Most have an average depth of sequence coverage of 2.3 × ; that is, each Y base pair in each individual was sequenced on average 2.3 times. However, two Y chromosomes were sequenced more deeply, to an average coverage of 26.2 × . The samples come from four worldwide populations representing three continental regions: 1) Yoruba in Ibadan, Nigeria from sub-Saharan Africa, abbreviated YRI; 2) CEPH (Centre d’Etude du Polymorphisme Humain)-Utah residents with ancestry from northern and western Europe, abbreviated CEU; 3) Han Chinese in Beijing, China, from East Asia, abbreviated CHB; and 4) Japanese in Tokyo, Japan, also from East Asia and abbreviated JPT. At the classification level used, ten different Y haplogroups were represented (Supplementary Table 1)(Supplementary Table 1). Aligned data in the form of BAM files were downloaded from the FTP site of the 1000 Genomes Project: *ftp://ftp.1000genomes.ebi.ac.uk/vol1/ftp/pilot_data/.*

In addition to the Y-CNVs discovered during the current study, in our final data set we included the only Y-deletion reported and validated in the original study of these genomes (24), and 4 Y-CNVs reported in a more recent study that used these and additional samples (7). Most of the analyses were performed from September to December 2010, and from January to July 2012, with follow-up and manuscript preparation August 2012 to November 2014.

### Complete Genomics data

A total of 8 male samples that are in common between 1000 Genomes Pilot 1 data and the Complete Genomics Public Data set (v36 v2.0.0) were analyzed. These samples were sequenced at an average depth of 25.4 × using 33 bp reads (Supplementary Table 1)(Supplementary Table 1). Sequencing data in the form of mapping.*.tsv.bz2 and read.*.tsv.bz2 files were downloaded and converted into BAM files using cgatools-1.4.0.15. Additionally, we also included insertions and deletions (>50 bp) reported in the high-quality release of Y-SVs on the same samples using the Complete Genomics Analysis Pipeline Version 2.0. All data were downloaded from the FTP site of Complete Genomics: *ftp://ftp2.completegenomics.com/.*

### OMNI SNP-chip data

Normalized SNP intensity data from Illumina HumanOmni2.5-8 arrays generated for the entire set of 1000 Genomes Project samples were analyzed (Supplementary Table 1)(Supplementary Table 1). Data were downloaded from the FTP site of the 1000 Genomes Project: *ftp://ftp.1000genomes.ebi.ac.uk/vol1/ftp/technical/working/20120131_omni_genotypes_and_intensities/.*

### DNA samples for experimental validation

Experimental validation of some SVs was carried out as described below using DNA samples obtained from the Coriell Institute for Medical Research, *http://www.coriell.org/.*

### Depth of coverage CNV analysis

CNVs were called using three different data sets. 1) Low-coverage (2.3 × ) samples with Illumina sequence data, 2) low-to-high coverage (4-23 × ) pools that were built by merging low-coverage samples from the same haplogroup (23), and 3) high-coverage (25.4 × ) samples from Complete Genomics. A read depth-of-coverage (DOC) analysis was implemented (25) on a total of 70, 10, and 8 samples for each data set, respectively (Supplementary Table 1)(Supplementary Table 1). This method follows a rationale derived from aCGH experiments: first, instead of hybridizing DNA against a microarray, sequence reads from two different samples are aligned to the same genome template. Second, instead of measuring levels of fluorescence, the number of reads in each sample is directly counted using a sliding-window approach. Finally, instead of searching for significant differences using intensity levels, differences in coverage between the two samples are calculated and then transformed into log2-ratios. Differences exceeding a significance threshold are indicative of relative gains or losses of genetic material.

The individual NA12891 (CEU trio father) was used as the reference for comparisons against all the other samples. A threshold (T) and a sliding-window size (WS) that best fitted each of our samples was chosen based on the following reasoning: we sub-sampled the reference individual NA12891 to a series of different coverage levels representative of the samples in each data set. We then performed DOC analysis between each of the sub-samples and the complete NA12891 reference. Since this last analysis compares samples from the same individual, we expected no CNVs to be detected. Combinations of T and WS values producing this expected result were chosen as the best for each data set (Supplementary Table 2)(Supplementary Table 2).

A problem often encountered when using DOC strategies is the fragmentation of CNVs into two or more adjacent segments that actually correspond to the same variant. This is due to local minima in the log-2 ratio signal that fail to meet the threshold established. To account for this, we joined into single calls all segments with an upstream or downstream neighbor separated by a distance of 5 kb or less (see Supplementary Table 3 (Supplementary Table 3) and Supplementary Table 4 (Supplementary Table 4) for a description of SV calls after and before merging, respectively). Using a modified version of the *cnv* R-package present in the CNV-seq method (25), we generated coverage plots for each of the detected CNV regions in all the samples. Finally, all CNVs showing weak evidence based on a visual inspection of these plots were discarded. A total of 16 CNVs were identified using this strategy (Supplementary Material, pages 1-26 (Supplementary material)).

### Paired-end analysis

Paired-end methodologies are typically used for the discovery of insertions and deletions via the identification of read pairs whose mapping position deviates significantly from the expected distance relative to a reference (1). This indicates that the read pair was derived from an individual whose structure differs from the reference sequence. We performed an analysis of discordant paired-end reads (26) in the three data sets previously described. Because not all the 1000 Genomes Pilot 1 Project samples were sequenced using paired-end reads, we only used samples and libraries that were sequenced using paired-end reads. All the Complete Genomics samples were sequenced using paired-end reads. Paired-end approaches rely heavily on the correct mapping of reads in the genome. We filtered out the reads with a mapping quality below 35 in all samples using samtools-0.1.15 (27). Overall this resulted in the use of 58 samples from the 1000 Genomes Pilot 1 Project, 8 from Complete Genomics, and 10 haplogroup pools. The average depth for the three data sets was 0.8 × , 26.1 × , and 4.1 × , respectively (Supplementary Table 1)(Supplementary Table 1). Given these differences in read depth, a minimum of 2 read pairs supporting an SV was required in the case of the 1000 Genomes Pilot 1 samples and 4 in the cases of Complete Genomics and haplogroup pools. For the rest of the parameters, default settings were used. Individual images of the SVs detected were created using the IGV viewer (28) and visually analyzed to filter out possible false positives. This strategy resulted in 5 deletions that were selected for polymerase chain reaction (PCR) validation.

### Validation using OMNI SNP-chip data

In order to search for additional supporting evidence for all the variants detected, we used normalized SNP intensity data from Illumina Omni 2.5 SNP-chip arrays, which is available for all the samples analyzed. We could thus investigate whether or not a candidate CNV was supported by independent copy number (ie, intensity) data. Samples not present in our analysis, as well as SNPs not present on the Y chromosome, were filtered out. This resulted in a total density of 1953 SNPs available for all the 70 samples previously analyzed. Since intensity data were present for both alleles of each SNP and we were interested in the overall intensity (indicative of copy number) for each SNP position, intensities for both alleles were summed into single values. As in the previous analyses, we used the sample NA12891 as a reference and calculated log2-ratios between SNP intensities in this sample and all the other samples. Individual plots for all samples and variants were generated using R (29) and deviations from 0 (no copy number difference) were compared with the log-2 ratio plots of the DOC analysis on the same variants.

### Experimental validation of large partial deletions in the AZFc region

Single PCR reactions using the sequence-tagged site (STS) markers sY1291, sY1191, sY1161, and a multiplex reaction including sY1206 and sY1201, were performed on all samples. The absence of the sY1291 product and the presence of the rest of the markers indicated the presence a *gr/gr* deletion (30), whereas the absence of sY1191 and the presence of the rest indicated a *b2/b3 (g1/g3)* deletion (31,32). All samples shown to carry either of these deletions were tested a second time using single PCR reactions for all the markers and also using the combination of singleplex and multiplex reactions. All results were successfully confirmed in this way. Singleplex and multiplex PCR reactions were performed using 50 ng genomic DNA template, 2-8 pmol of each primer in 50 mM KCl, 10 mM Tris-HCl (pH 8.3), 1.5 mM MgCl_2_, 0.1% Triton-X100, 200 µM of each dNTP, and 1 unit of Taq polymerase (Promega, Madison, WI, USA) in a final volume of 20 µL. Amplification cycles consisted of an initial denaturation step at 94°C for 4 min, plus 35 cycles at 94°C for 30 s, annealing at 57°C, 61°C, 58°C (each for 45 s) and 72°C for 45 s, and a final extension of 72°C for 5 min. Reaction products were analyzed by agarose gel electrophoresis.

### Experimental validation of deletions <10 kb

Single PCR reactions were performed on all deletions smaller than 10 kb that had no previous experimental validation. Primer3 (33) was used for designing the primers; these were located at a distance between 200-1000 bp outside the detected start and end of the variants. *In-silico* PCR and RepeatMasker tools from the UCSC Browser (34) were used to detect cases in which primers were predicted to generate more than one amplicon and/or were placed in highly repeated regions of the genome; such primers were avoided. PCRs were performed according to the length of the deletion in two different ways. 1) For deletions shorter than 1 kb, we used 10 ng of genomic DNA template, 5 pmol of each primer in 50 mM KCl, 10 mM Tris-HCl (pH 8.3), 3.5 mM MgCl_2_, 0.01% (w/v) gelatin, 250 µM of dNTPs, and 0.45 units of Taq polymerase (Applied Biosystems, Life Technologies, Waltham, MA, USA) in a final volume of 20 µL. Amplification cycles consisted of an initial denaturation step at 94°C for 4 min, plus 35 cycles at 94°C for 30 s, annealing at 57°C, 61°C, 58°C (each for 45 s) and 72°C for 45 s, and a final extension of 72°C for 5 min. 2) For deletions greater than 1 kb, we used 50 ng genomic DNA template, 10 pmol of each primer in 50 mM KCl, 10 mM Tris–HCl (pH 8.3), 1.5 mM MgCl_2_, 0.1% Triton-X100, 200 µM of each dNTP, and 1 unit of Taq polymerase in a final volume of 20 µL. Amplification consisted of an initial denaturation step at 94°C for 15 min, plus two rounds of 13 cycles each. The first one at 94°C for 30 s, annealing at 68°C for 30 s and decreasing 0.5°C each cycle, and extension at 68°C for 10 min. The second one at 94°C for 30 s, annealing at 58°C for 30 s, and extension at 68°C for 10 min. Reaction products were analyzed using agarose gel electrophoresis. SVs that failed validation are also reported (Supplementary Table 3 (Supplementary Table 3) and Supplementary Table 4(Supplementary Table 4)).

### Data integration

Evidence from all analyses and data sources (8 in total) were integrated into a highly curated data set. All SVs reported in this analysis were supported by at least two different lines of evidence (Table 1). A full description of all SVs, including information from the multiple sources of evidence, is provided in Supplementary Table 3(Supplementary Table 3). Y-chromosomal haplogroup analysis was based on the ISOGG Y-DNA Haplogroup Tree 2015 (*http://www.isogg.org/tree/ISOGG_YDNATreeTrunk.html*) and sub-branches.

**Table 1:** Summary of the 19 validated structural variants (SVs)*

SV ID	Chromosome	Region start	Region end	Type of event	Length (bp)	# Samples	MSY Class	SDs present in the region	Main genes	Data set	Comment	P1T	CGT	CGR	P1R	Ph1R	OCT	L	PCR	# Sources of validation	Sources of validation
SV 01	Y	3.109.266	3.111.300	Gains:0,Deletions:4	2.034	4	X-Transposed	+	NA	Phase 1 report	NA	-	-	-	-	+	-	-	+	2	Platform(1),PCR
SV 02	Y	6.543.750	6.573.750	Gains:22,Deletions:0	30.000	22	X-Transposed	+	NA	This work	New	+	+	-	-	-	-	-	-	2	Platforms(2+)
SV 03	Y	7.761.250	8.001.250	Gains:1,Deletions:0	240.000	1	Ampliconic	-	NA	This work	New	+	-	-	-	-	+	-	-	2	Platforms(2+)
SV 04	Y	9.172.875	9.236.625	Gains:4,Deletions:1	63.750	5	Ampliconic	+	*TSPY*	This work	*TSPY* array	+	+	-	-	-	+	+	-	4	Platforms(2+),Literature
SV 05	Y	9.300.125	9.367.625	Gains:6,Deletions:0	67.500	6	Ampliconic	+	*TSPY*	This work	*TSPY* array	+	-	-	-	-	+	+	-	3	Platforms(2+),Literature
SV 06	Y	9.639.875	9.650.375	Gains:0,Deletions:8	10.500	8	Ampliconic	+	*TTTY22*	This work	NA	+	+	+	-	-	+	-	-	4	Platforms(2+)
SV 07	Y	10.016.250	10.041.250	Gains:1,Deletions:36	25.000	37	Heterochromatic	+	NA	This work	Alphoid Repeats	+	-	-	-	-	-	+	-	2	Platform(1),Literature
SV 08	Y	10.083.750	10.104.553	Gains:0,Deletions:13	20.803	13	Heterochromatic	-	NA	This work	Alphoid Repeats	+	+	-	-	-	+	+	-	4	Platforms(2+),Literature
SV 09	Y	13.104.553	13.126.250	Gains:0,Deletions:17	21.697	17	Heterochromatic	-	NA	This work	Repeats	+	+	-	-	-	+	+	-	4	Platforms(2+),Literature
SV 10	Y	13.136.250	13.143.954	Gains:0,Deletions:9	7.704	9	Heterochromatic	-	NA	This work	Repeats	+	-	-	-	-	-	+	-	2	Platform(1),Literature
SV 11	Y	13.446.250	13.688.750	Gains:4,Deletions:38	242.500	40	Heterochromatic	+	NA	This work	Repeats	+	-	+	-	-	-	+	-	3	Platforms(2+),Literature
SV 12	Y	14.208.831	14.208.912	Gains:0,Deletions:4	81	4	X-Degenerate	-	NA	Pilot 1 report	NA	-	-	-	+	-	-	-	+	2	Platform(1),PCR
SV 13	Y	17.306.559	17.311.584	Gains:0,Deletions:3	5.025	3	X-Degenerate	+	NA	This work	NA	+	-	-	-	+	+	-	+	4	Platforms(2+),PCR
SV 14	Y	22.223.737	22.434.987	Gains:25,Deletions:39	211.250	64	Heterochromatic	-	NA	This work	*DIZ19*	+	+	+	-	+	-	-	-	4	Platforms(2+)
SV 15	Y	22.464.987	22.471.737	Gains:11,Deletions:3	6.750	14	Heterochromatic	-	NA	This work	*DIZ19*	+	-	+	-	+	-	-	-	3	Platforms(2+)
SV 16	Y	24.875.619	26.526.445	Gains:3,Deletions:9	1.650.826	12	Ampliconic	+	*BPY2,DAZ1, DAZ2,PRYP3, CDY1B*	This work	gr/gr deletion	+	+	+	-	-	+	+	+	6	Platforms(2+),Literature, PCR
SV 17	Y	25.299.362	25.424.362	Gains:14,Deletions:9	125.000	19	Ampliconic	+	*DAZ1,DAZ2*	This work	*DAZ 1-2*	+	+	+	-	-	-	+	-	4	Platforms(2+),Literature
SV 18	Y	25.505.069	27.435.593	Gains:0,Deletions:3	1.930.524	3	Ampliconic	+	*PRYP3,CDY1B, BPY2B,DAZ3, DAZ4*	This work	*b2/b3(g1/g3)* deletion	-	-	-	-	-	-	+	+	2	Platform(1),Literature, PCR
SV 19	Y	26.929.362	27.046.862	Gains:15,Deletions:10	117.500	19	Ampliconic	+	*DAZ3,DAZ4*	This work	*DAZ 3-4*	+	+	+	-	-	-	+	-	4	Platforms(2+),Literature

*Type of event: total number of deletions and duplications that carry the variant. MSY Class: type of Y sequence class in which the SVs were located. Segmental duplications (SDs) associated in the Region: + = overlapping or flanking segmental duplications around the SV. Genes: protein-coding genes that overlap each SV. Data set: source of each SV. Comment: relevant prior information for each SV; variants that have not been previously reported elsewhere are labeled as “New”. P1T: supporting evidence from the analysis of Pilot 1 Data on this work. CGT: supporting evidence from the analysis of Complete Genomics Data as part of this work. CGR: supporting evidence from previously reported data for Complete Genomics samples. P1R: supporting evidence from previously reported data for Pilot 1 samples. Ph1R: supporting evidence from previously reported data on 1000 Genomes Phase 1 sample. OCT: supporting evidence from the analysis of OMNI SNP arrays as part of this work. L: supporting evidence from information in published literature. Polymerase chain reaction (PCR): supporting evidence from PCR experiments performed as part of this work. # Sources of Validation: total number of independent sources of validation. Sources of Validation: “Platform(1)” corresponds to the presence of evidence from one genomic technology and “Platform(2+)” indicates the presence of supporting evidence from two or more genomic technologies; literature and PCR evidence are considered to be separate. For all columns containing “+” or “-” symbols, the former indicates “presence” and the latter “absence”.

## Results

We applied a combination of read-depth and read-pair methods to discover SVs in 70 Y chromosomes from Africa, Europe, and East Asia with both low and high sequence coverage levels. Our strategy included the pooling of closely-related samples by haplogroup in order to increase the number of high coverage samples in the data set. We then used comparisons between Illumina and Complete Genomics sequencing, SNP-chip data, established and novel PCRs, and compiled literature reports in order to validate and support our set of calls. This combined strategy resulted in a set of 19 highly-curated and validated SVs (Supplementary Material, pages 1-26 (Supplementary material)) (Table 1).

**Figure 1:**
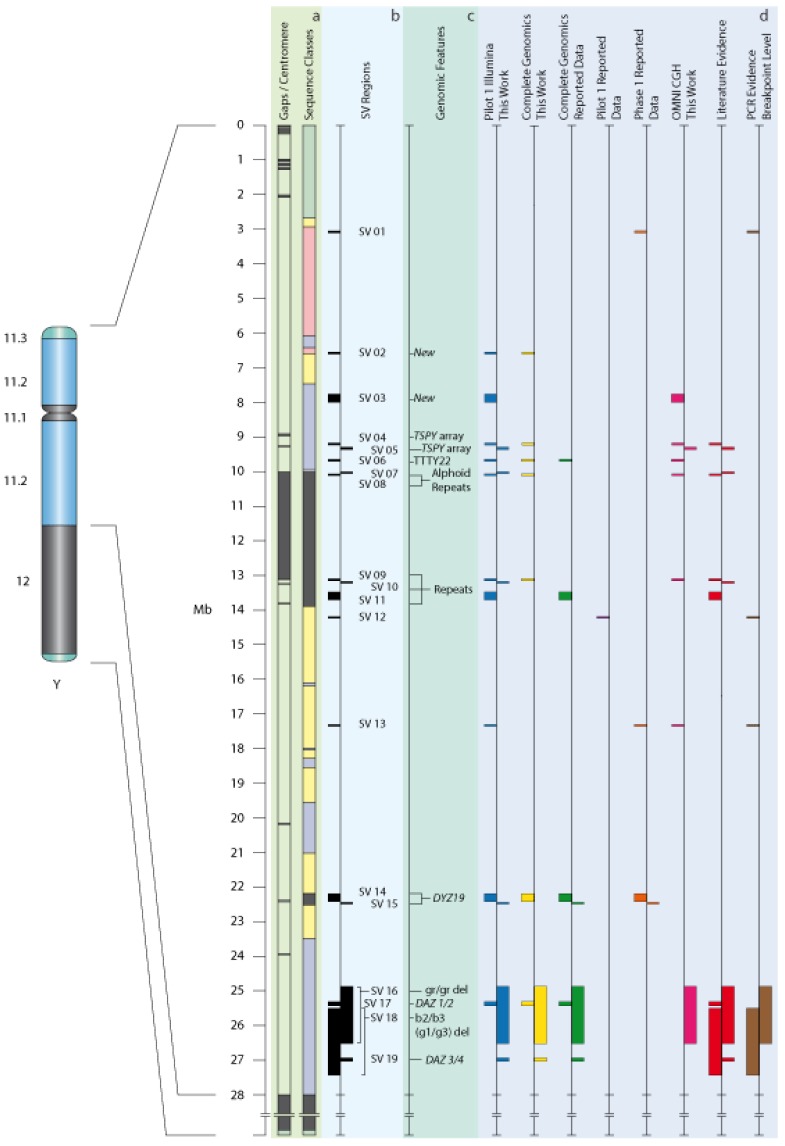
Schematic representation of the Y chromosome and the structural variants (SVs) detected. Y-chromosome left bar: the two extreme tips in green correspond to the two pseudoautosomal regions, and the rest of the chromosome, made up of two blue (euchromatic) and dark-gray (heterochromatic) sections, to the male-specific region. (**A**) Gaps/centromere: all dark-gray bars indicate gaps in the Y reference including the long centromeric region at ~ 10-13 Mb. Sequence classes: pseudoautosomal (green), X-degenerate (light-yellow), X-transposed (light-pink), ampliconic (light-blue), and heterochromatic (dark-gray) regions are indicated along the chromosome. (**B**) SV locations; approximate locations of the 19 SVs described in this work. (**C**) Genomic features. Relevant previous information available about some SVs; SVs not previously described are labeled as “New”. (**D**) The eight tracks shown in this section indicate the different sources of evidence for each of the SVs. In all cases (A, B, C, and D) the regions beyond ~ 28 Mb (shown in panel a) as gray and green blocks) correspond to heterochromatic regions variable in size, and the pseudoautosomal region in the long arm of the Y, respectively.

The SVs are numbered SV 01 to SV 19 according to their location on the chromosome (Figure 1, Table 1). Of this total, our methodology was able to detect 16 SVs, compared to 6, 1, and 4 that were reported in the same samples by Complete Genomics, Pilot 1 (23) and Phase 1 of the 1000 Genomes Project (24), respectively. Likewise, 8 SVs are unique to our analysis, compared to 0, 1, and 1 to the other reports (Table 1). Importantly, 2 of the 16 new SVs have not been reported and validated before. These numbers highlight the low level of attention paid to the Y chromosome by previous studies of these samples, coupled with the difficulty of correctly identifying large CNVs in the repeat-rich Y chromosome.

**Figure 2:**
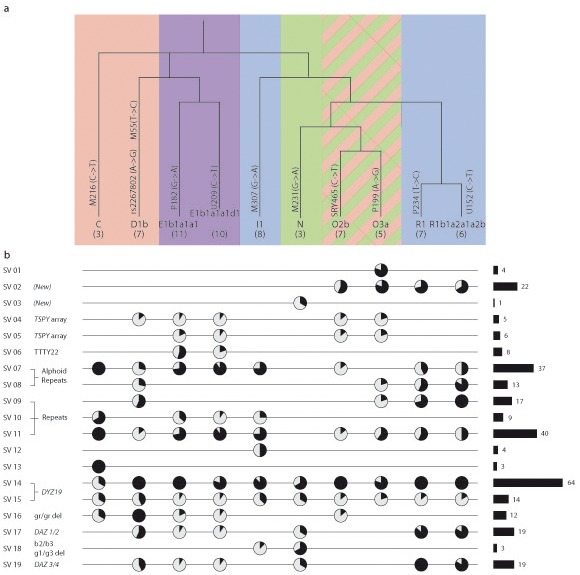
Phylogenetic framework for the study of Y-structural variants (SVs). (**A**) Branches of the Y phylogeny present in this work. Haplogroup names and number of samples within each branch are shown at the bottom of the panel. Background colors represent the population that each of the haplogroups belongs to; red indicates JPT, purple YRI, blue CEU, and green CHB (acronyms are explained in the Methods). Haplogroups O2b and O3a contain samples that belong to both JPT and CHB, indicated by the green and red stripes. (**B**) Each row represents one of the 19 SVs reported in this work. There are four sections of information for each row. From left to right: 1) ID of the variant. 2) Relevant information available for the variant. 3) Pie charts are shown for all haplogroups that carry the variant. Black areas within these pie charts represent the proportion of samples containing the variant compared with the total number of individuals in each haplogroup. 4) Horizontal black bars on the right of the panel show the total number of individuals that carry the variant.

Our data set contains SVs with sizes ranging from 81 bp to 1.9 Mb. Of the 19 SVs detected, 14 were larger than 10 kb, 4 were between 5 kb and 10 kb, and one was below 100 bp. A bias toward identifying large SVs is evident, and the approaches based on read-depth that are more sensitive to large events stand out for their efficacy in identifying such events, with 16 SVs being detected, in comparison to only one using paired-end approaches. In terms of the nature of the events, a slight bias toward deletions was observed: 12 of the SVs identified showed a higher proportion of deletions vs duplications among samples, with 8 SVs showing no duplication signal at all. Non-reference allele counts ranged between one and 64 among the 70 Y chromosomes. SVs with the highest observed non-reference allele frequencies were largely but not entirely concentrated in the heterochromatic segments near the centromere and *DYZ19* locus. In contrast, low-frequency SVs, among which one is novel, were more associated with X-degenerate and ampliconic segments. Since the haplogroup assignments of all the Y chromosomes studied here have previously been identified (23) (Figure 2), this information is used below, along with location, size, and frequency, in presenting each SV.

**Figure 3:**
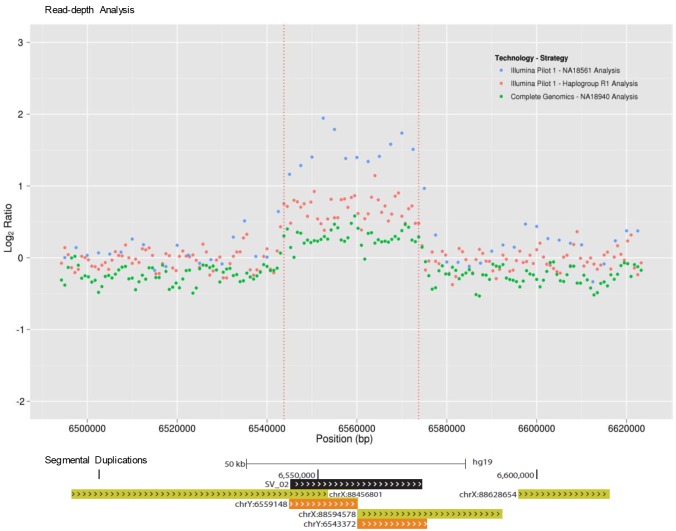
Evidence supporting the duplication structural variant (SV) 02. From top to bottom: 1) Read-depth analysis. Read-depth plots for Pilot 1 sample NA18561 (blue), Pilot 1 pooled sample Haplogroup R1 (red), and Complete Genomics sample NA18940 (green). 2) Segmental duplications (from the UCSC genome browser). Bars colored in gray, dark-yellow, and dark-orange correspond to duplications with 90%-98%, 98%-99%, and >99% sequence identity, respectively. All comparisons in the read-depth plots are expressed in log2-ratios and use the reference individual NA12891. Red vertical dotted lines indicate the approximate start and end positions of the SV (see Table 1).

Two SVs were found to overlap with the X-transposed regions of the Y: SV 01 was reported in the Phase 1 release and corresponded to a high-frequency 2 kb deletion present only in haplogroup O3a-P199 (Figure 2). Although we did not discover this variant using our approaches, we successfully validated it using PCR (Supplementary Material, page 2 (Supplementary material)). SV 02 corresponded to a large (30 kb) and previously undescribed duplication present in 22 individuals from the CEU, CHB, and YRI populations within haplogroups O and R. This duplication is located at the breakpoints of two segmental duplications with high levels of sequence identity (>99%) and has evidence from both low and high coverage samples as well as from the pooling of individuals in haplogroup R1-P234 (Figure 3). The specific Y-haplogroup distributions of these two SVs provide strong evidence for their location on the Y-chromosomal, rather than X-chromosomal copy of this transposed region.

**Figure 4:**
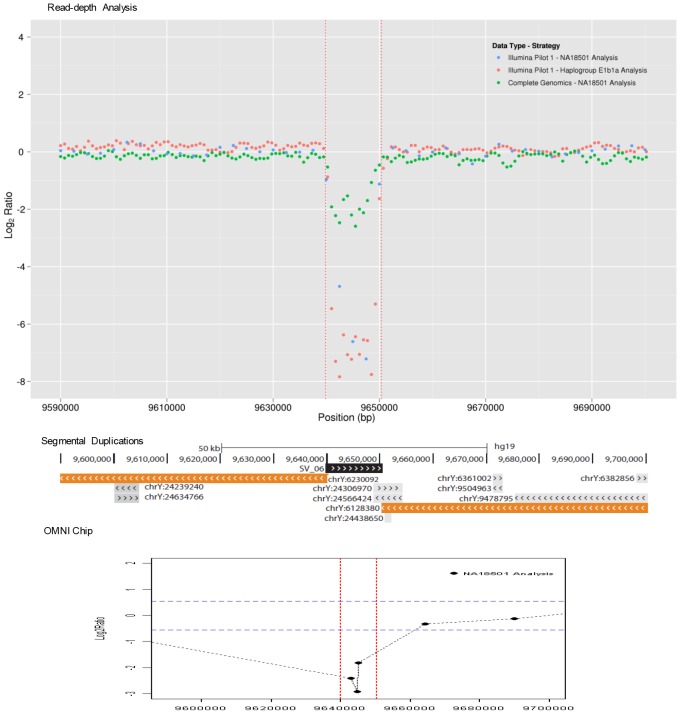
Evidence supporting the deletion structural variant (SV) 06. From top to bottom: 1) Read-depth analysis. Read-depth plots for Pilot 1 sample NA18501 (blue), Pilot 1 pooled sample Haplogroup E1b1a1a1 (red), and Complete Genomics sample NA18501 (green). 2) Segmental duplications. Bars colored in gray, dark-yellow, and dark-orange correspond to duplications with 90%-98%, 98%-99%, and >99% sequence identity, respectively. 3) OMNI Chip data. Single nucleotide polymorphism (SNP) intensities of sample NA18501 at the variant location. Blue horizontal lines are positioned at log2-ratios -0.6 and 0.6. All comparisons in the read-depth and SNP intensity plots are expressed as log2-ratios and use the reference individual NA12891. Red vertical dotted lines indicate the start and end positions of the SV (Table 1).

Eight SVs were found in the ampliconic segments: SV 03 corresponded to a large (240 kb) and previously undescribed SV present only in one individual belonging to haplogroup N-M231. SVs 04 and 05 corresponded to variation in the *TSPY* arrays on the Y. Only the *TSPY* array overlapping SV 05 was previously known to be variable (13,14,17), but since the two arrays are comprised of the same repetitive sequences, read mapping is expected to occur equally well at both (“shadowing”), thus making the array overlapping SV 04 appear variable as well. SV 06 overlapped the non-coding RNA *TTTY22* and corresponded to a 10 kb deletion present only in YRI individuals within haplogroup E, and was previously reported only in the Complete Genomics release but not validated by other sources until now. This deletion is also located at the breakpoints of two segmental duplications with high levels of sequence identity (>99%) and is supported by evidence from both low and high coverage samples as well as from the pooling of individuals in haplogroup E1b1a1a1-P182; validation by SNP-chip data was also observed (Figure 4). SVs 16 and 18 corresponded to the well-known large *gr/gr* and *b2/b3 (g1/g3)* deletions, respectively (30-32). SVs 17 and 19 mapped to the well-known DAZ-repeat regions within the *DAZ 1-2* and *DAZ 3-4* genes (35), respectively. These four SVs (16-19) were present at high frequency across all populations studied, with the exception of SV 18, which was present at low frequency in the CEU and CHB populations only. They were all present in multiple haplogroups, and most were variable within each haplogroup, indicating their multiple origins, although the previously observation of fixation of the *gr/gr* deletion in haplogroup D (30) was replicated here.

Seven SVs were present in the heterochromatic regions forming three different groups: the first group (SVs 07 and 08) was located in the periphery of the centromeric region on the short arm of the chromosome. These variants corresponded to regions rich in alphoid repeats and were present at high frequency. The second group (SVs 09, 10, and 11) was located in the opposite edge of the centromere, on the long arm. These regions corresponded to repeats of variable length and also showed high-frequency variation. The third group (SVs 14 and 15) was located on the long arm of the chromosome and corresponded to the highly variable region *DYZ19*. SV 14 in fact was the most variable region in this study, with non-reference structures detected in 64 samples.

**Figure 5:**
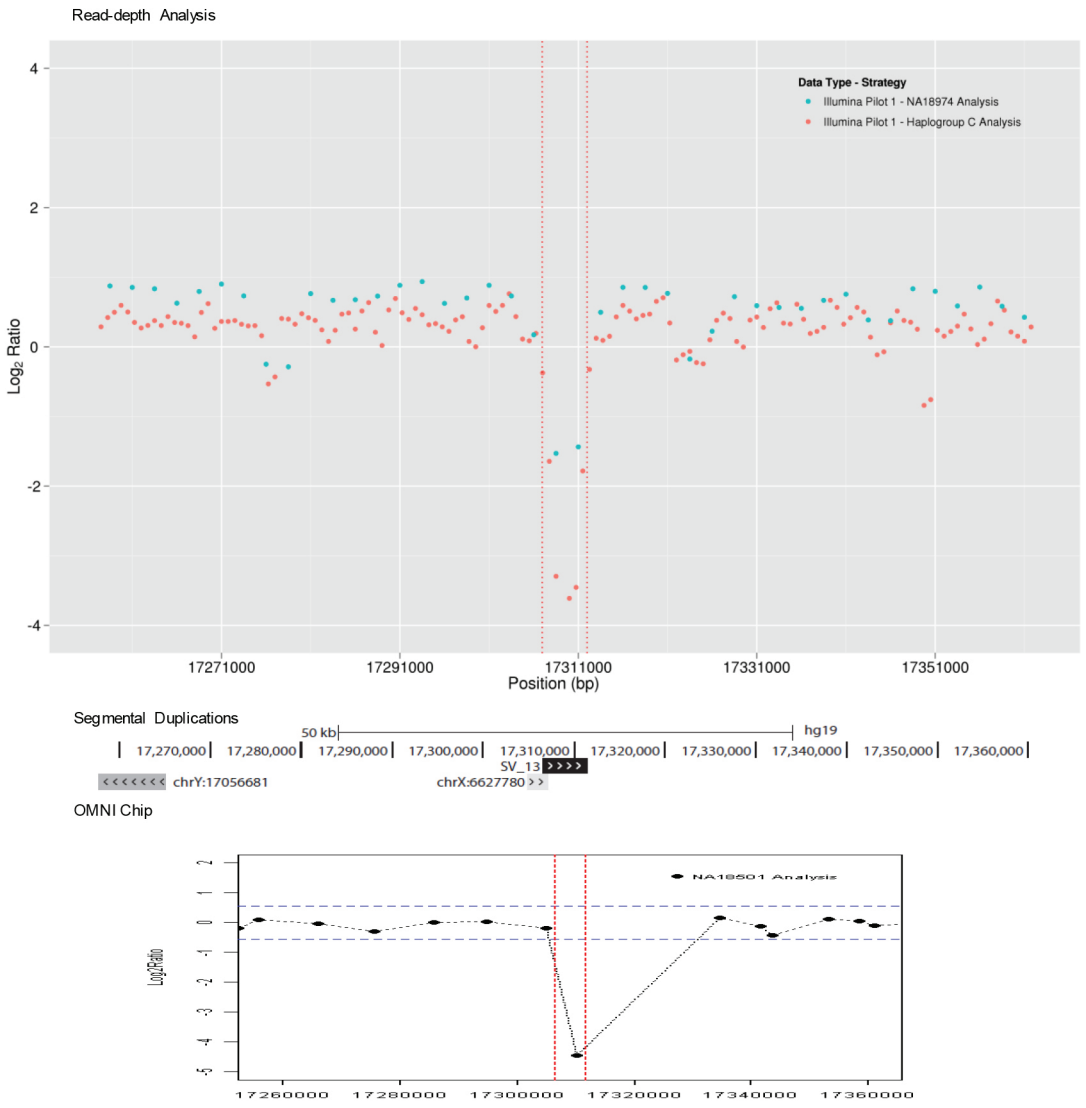
Evidence supporting the deletion structural variant (SV) 13. From top to bottom: 1) Read-depth analysis. Read-depth plots for Pilot 1 sample NA18974 (blue) and Pilot 1 pooled sample from Haplogroup C (red). 2) Segmental duplications. Bars colored in gray correspond to duplications with 90%-98% sequence identity. 3) OMNI Chip data. Single nucleotide polymorphism (SNP) intensities of sample NA18501 at the variant location. Blue horizontal lines are positioned at log2-ratios -0.6 and 0.6. All comparisons in the read-depth and SNP intensity plots are expressed in log2-ratios and use the reference individual NA12891. Red vertical dotted lines indicate start and end positions of the variant (Table 1).

Finally, 2 SVs were found in the X-degenerate regions of the Y, both at very low frequency: SV 12 is an 81 bp deletion only present in 4 individuals from the European I1-M307 haplogroup and corresponded to the only region reported and validated using PCR by the Pilot 1 release (24). It also was the only SV in our validated set detected using paired-end approaches. SV 13 is a 5kb deletion present in all three samples from the Japanese haplogroup C-M216 (including the pooled sample for this haplogroup), and is specific to this haplogroup. PCR validation was successfully conducted on this variant (Supplementary Material, page 18 (Supplementary material)). This variant was not associated with segmental duplications at the breakpoints and the one SNP overlapping the region was found to support the deletion in all deleted samples (Figure 5).

At the population level, the highest variability was found in YRI samples, whereas the lowest was in individuals from the CHB population. We also found that 12 (63%) of the SVs detected overlapped segmental duplications (SDs). Roughly 36% of the Y chromosome is composed of SDs (36), so the proportion of SVs observed to overlap SDs is larger than expected for a random distribution.

The Y chromosome codes for only 27 distinct proteins, although the genes for several of these are present in multiple copies (36). Despite this very low gene density, 6 of the 19 SVs (SVs 04-05, 16-19) overlapped with 5 of the gene families (*BPY, CDY, DAZ, PRY, TSPY*), and a seventh SV (SV 06) with the long non-coding RNA *TTTY22*.

## Discussion

The Y chromosome has been under-represented in recent studies of human SVs, particularly those based on sequence data, where a total of just 5 Y-SVs were reported in two large studies (7,24); moreover, all those Y-SVs already known to be present at high frequencies in some or all of the populations investigated (5,13,15,16) were not called in the sequence-based studies, although their large sizes should have made them readily detectable. Here, we showed that all of these common known SVs can be successfully identified using current data when appropriate analysis methods are applied.

Our findings emphasize the major roles of both highly-repeated heterochromatic regions and also segmental duplications in providing a sequence environment for SV generation. The surrounding sequence and haplogroup distribution can provide insights into the mutational mechanisms that may have generated these Y-SVs (8). If the surrounding sequences are repeated, non-allelic homologous recombination (NAHR) may occur, duplicating or deleting the region between the repeats; if they are not repeated, SVs may be generated by a number of other mechanisms, the most relevant of which here is non-homologous end joining (NHEJ). NAHR-generated SVs are quite likely to recur, and duplications can revert, while NHEJ-generated SVs are less likely to do either. The well-established Y-chromosomal phylogeny (22) allows us to ask whether SV haplogroup distributions are consistent with a single mutational event, or require more than one event, such as recurrence of the same mutation or reversion, to explain their distribution. For example, SV 01, where all four variant alleles lie within haplogroup O3a-P199 (Figure 2) is consistent with a single mutational origin. Moreover, this origin can be placed in time after the mutation that created the SNP defining haplogroup O3a-P199. In contrast, SV 15, where both reference and non-reference alleles are found in all haplogroups examined here (Figure 2) must have experienced at least 10 mutational events. Applying this reasoning to all 19 SVs shows that just four (SVs 01, 03, 12, and 13) could have arisen by single mutations, while all the others have more complex multi-mutational origins. Furthermore, these four variants do not show any segmental duplication structure surrounding the breakpoints, in concordance with a single mutational origin associated with NHEJ. In contrast, 11 of the remaining 16 SVs are associated with SDs and are thus likely to have arisen by NAHR. In some cases we can infer the likely sequence of mutations in more detail. For example, SV 02 occurs only in the related haplogroups O2b-SRY465, O3a-P199, R1-P234, and R1b1a2a1a2b-U152, yet both reference and duplicated alleles occur in all four haplogroups. Its absence from the 42 chromosomes belonging to other haplogroups suggests that its origin (ie, the duplication event) may be rare, and have occurred only once, in the common ancestor of haplogroups O and R. But the occurrence of reference alleles in haplogroup N and each of the four O and R haplogroups suggests that reversions to the reference state are more frequent, and have occurred at least five times, ie, once in each haplogroup.

The observation that 6 (SVs 04, 05, 16-19) of the 19 SVs affect the copy number of 5 gene families is particularly striking in the context of the very low gene density on the Y chromosome. These SVs are relatively common, with non-reference allele counts ranging from 3 (SV 18) to 19 (SVs 17 and 19) of the 70 Y-chromosomes. Several factors contribute to this situation. First, several of the SVs are large, two duplicating or deleting 1.6 and 1.9 Mb of the 24 Mb of the male-specific Y euchromatin, so there is an increased chance of them affecting genes. Second, each of the SVs probably affects only some members of any gene family, although for SVs 17 and 19, which both reflect differences in copy number of the DAZ repeat domain, this is uncertain because of the shadowing effect mentioned earlier. Third, the known functions of all of the 5 genes are linked to spermatogenesis, a phenotype which is variable in the population and where duplication or deletion of a few of the contributing genes may have only minor effects; indeed, only the *gr/gr* deletion (SV 16) and *TSPY* copy number variation (SVs 04 and 05) have been linked to a slightly increased risk of spermatogenic failure in some populations (19,30), while the *b2/b3(g1/g3)* deletion (SV 18) and DAZ repeat variation (SVs 17 and 19) appear to be neutral (31,32,35). The phenotypes of the 1000 Genomes donors are unknown, but our findings suggest that the 5 *gr/gr* deletion carriers outside haplogroup D and the individuals with the lowest *TSPY* copy number may be at increased risk of spermatogenic impairment.

This survey of Y-chromosomal SVs is likely to be complete for large common SVs in the haplogroups included in these population samples and the regions of the chromosome accessible to current sequencing technology. Nevertheless, populations carry far more small rare SVs (7), and so the survey is likely to be very incomplete for this class. SVs smaller than 5 kb were not detected by the major discovery approach used here, read depth, although 2 are included in the data set, discovered in other ways (7,23) and 5 more candidates were detected by our paired-end analysis but all turned out to be false positives after PCR testing. Despite the limitation in discovering small CNVs from the low-coverage sequence data used, this work demonstrates the power of using sequence data for SV discovery and analysis on the Y chromosome, and points to the need for larger and more comprehensive surveys. Indeed, the 1000 Genomes Project has itself increased the sequence coverage and sample size considerably, so such improved studies are now possible.
