# Supplementary material for: Structural variation on the human Y chromosome from population-scale resequencing
Source: Croat Med J. 2015 Jun;56(3):194–207. doi: 10.3325/cmj.2015.56.194 (PMC4500966; doi:10.3325/cmj.2015.56.194)
Supplement: Supplementary Table 1 [file CroatMedJ_56_s001.pdf]

OMNI_Chip_Data				
Coriell_Sample_ID	CGH_Technology	Population	Haplogroup	#SNPs_MSY_Region
NA12003	Illumina HumanOmni2.5-8	CEU	I	1953
NA06994	Illumina HumanOmni2.5-8	CEU	I1	1953
NA07051	Illumina HumanOmni2.5-8	CEU	I1	1953
NA11829	Illumina HumanOmni2.5-8	CEU	I1	1953
NA11881	Illumina HumanOmni2.5-8	CEU	I1	1953
NA11919	Illumina HumanOmni2.5-8	CEU	I1	1953
NA11992	Illumina HumanOmni2.5-8	CEU	I1	1953
NA12750	Illumina HumanOmni2.5-8	CEU	I1	1953
NA12891	Illumina HumanOmni2.5-8	CEU	I1	1953
NA12155	Illumina HumanOmni2.5-8	CEU	R	1953
NA10851	Illumina HumanOmni2.5-8	CEU	R1	1953
NA11831	Illumina HumanOmni2.5-8	CEU	R1	1953
NA12043	Illumina HumanOmni2.5-8	CEU	R1	1953
NA12045	Illumina HumanOmni2.5-8	CEU	R1	1953
NA12154	Illumina HumanOmni2.5-8	CEU	R1	1953
NA12716	Illumina HumanOmni2.5-8	CEU	R1	1953
NA06986	Illumina HumanOmni2.5-8	CEU	R1b1a2a1a2b1b2	1953
NA07347	Illumina HumanOmni2.5-8	CEU	R1b1a2a1a2b1b2	1953
NA07357	Illumina HumanOmni2.5-8	CEU	R1b1a2a1a2b1b2	1953
NA11994	Illumina HumanOmni2.5-8	CEU	R1b1a2a1a2b1b2	1953
NA12005	Illumina HumanOmni2.5-8	CEU	R1b1a2a1a2b1b2	1953
NA12144	Illumina HumanOmni2.5-8	CEU	R1b1a2a1a2b1b2	1953
NA18558	Illumina HumanOmni2.5-8	CHB	N	1953
NA18608	Illumina HumanOmni2.5-8	CHB	N	1953
NA18562	Illumina HumanOmni2.5-8	CHB	O	1953
NA18603	Illumina HumanOmni2.5-8	CHB	O	1953
NA18605	Illumina HumanOmni2.5-8	CHB	O	1953
NA18638	Illumina HumanOmni2.5-8	CHB	O	1953
NA18561	Illumina HumanOmni2.5-8	CHB	O2b	1953
NA18563	Illumina HumanOmni2.5-8	CHB	O2b	1953
NA18572	Illumina HumanOmni2.5-8	CHB	O3a	1953
NA18609	Illumina HumanOmni2.5-8	CHB	O3a	1953
NA18971	Illumina HumanOmni2.5-8	JPT	C	1953
NA18974	Illumina HumanOmni2.5-8	JPT	C	1953
NA18940	Illumina HumanOmni2.5-8	JPT	D1b	1953
NA18944	Illumina HumanOmni2.5-8	JPT	D1b	1953
NA18948	Illumina HumanOmni2.5-8	JPT	D1b	1953
NA18952	Illumina HumanOmni2.5-8	JPT	D1b	1953
NA18960	Illumina HumanOmni2.5-8	JPT	D1b	1953
NA18961	Illumina HumanOmni2.5-8	JPT	D1b	1953
NA18967	Illumina HumanOmni2.5-8	JPT	D1b	1953
NA18943	Illumina HumanOmni2.5-8	JPT	O2b	1953
NA18953	Illumina HumanOmni2.5-8	JPT	O2b	1953
NA18965	Illumina HumanOmni2.5-8	JPT	O2b	1953
NA19005	Illumina HumanOmni2.5-8	JPT	O2b	1953
NA18945	Illumina HumanOmni2.5-8	JPT	O3a	1953

NA18959	Illumina HumanOmni2.5-8	JPT	O3a	1953
NA18486	Illumina HumanOmni2.5-8	YRI	E	1953
NA18856	Illumina HumanOmni2.5-8	YRI	E	1953
NA19239	Illumina HumanOmni2.5-8	YRI	E	1953
NA18504	Illumina HumanOmni2.5-8	YRI	E1b1a1a1	1953
NA18507	Illumina HumanOmni2.5-8	YRI	E1b1a1a1	1953
NA18510	Illumina HumanOmni2.5-8	YRI	E1b1a1a1	1953
NA18516	Illumina HumanOmni2.5-8	YRI	E1b1a1a1	1953
NA18519	Illumina HumanOmni2.5-8	YRI	E1b1a1a1	1953
NA18522	Illumina HumanOmni2.5-8	YRI	E1b1a1a1	1953
NA19098	Illumina HumanOmni2.5-8	YRI	E1b1a1a1	1953
NA19171	Illumina HumanOmni2.5-8	YRI	E1b1a1a1	1953
NA19200	Illumina HumanOmni2.5-8	YRI	E1b1a1a1	1953
NA19207	Illumina HumanOmni2.5-8	YRI	E1b1a1a1	1953
NA19210	Illumina HumanOmni2.5-8	YRI	E1b1a1a1	1953
NA18498	Illumina HumanOmni2.5-8	YRI	E1b1a1a1d1	1953
NA18501	Illumina HumanOmni2.5-8	YRI	E1b1a1a1d1	1953
NA18853	Illumina HumanOmni2.5-8	YRI	E1b1a1a1d1	1953
NA18871	Illumina HumanOmni2.5-8	YRI	E1b1a1a1d1	1953
NA19119	Illumina HumanOmni2.5-8	YRI	E1b1a1a1d1	1953
NA19138	Illumina HumanOmni2.5-8	YRI	E1b1a1a1d1	1953
NA19144	Illumina HumanOmni2.5-8	YRI	E1b1a1a1d1	1953
NA19153	Illumina HumanOmni2.5-8	YRI	E1b1a1a1d1	1953
NA19160	Illumina HumanOmni2.5-8	YRI	E1b1a1a1d1	1953

Samples_Analysed
#Samples
70

Samples_Analyzed	
Population	#Individuals
CEU	22
CHB	10
JPT	15
YRI	23
Total	70

Samples_Analyzed
#Haplogroups
7

Sample_Used_as_Reference			
Coriell_Sample_ID	Sequencing_Strategy	Population	Haplogroup
NA12891	Illumina HumanOmni2.5-8	CEU	I1
