# Supplementary material for: Structural variation on the human Y chromosome from population-scale resequencing
Source: Croat Med J. 2015 Jun;56(3):194–207. doi: 10.3325/cmj.2015.56.194 (PMC4500966; doi:10.3325/cmj.2015.56.194)
Supplement: Supplementary Table 2 [file CroatMedJ_56_s002.pdf]

**Supplementary_Table_4**
**Description**
This table contains the different log-2 thresholds (LT) and window sizes (WS) used in the study.

| Dataset / Platform | Sub-dataset | Samples / Haplogroup Pools | Log-2 ratio threshold | Window size | Reference |
|---|---|---|---|---|---|
| Low coverage Illumina | Low-coverage (2.3x) | NA12003,NA06994,NA07051,NA11829,NA11881,NA11919,NA11992,NA12750,NA12155,NA10851,NA11831,NA12043,NA12045,N | 1 | 10 kb | Low coverage NA12891 (4.2x) |
| | YRI Trio father (26.1x) | NA19239 | 0,6 | 3 kb | High coverage NA12891 (26.3x) |
| Low-to-high coverage Illumina | Low coverage (3.9x - 4.3x) | N and C | 1 | 10 kb | Low coverage NA12891 (4.2x) |
| | High coverage (8.4x - 22.8x) | O3e, R1b1b2, O2b, D, R1, E1b1a, I1, and E1b1a8a | 0,6 | 3 kb | High coverage NA12891 (26.3x) |
| High coverage Complete Genomics | High coverage (20.3x - 27.7x) | NA06994,NA10851,NA07357,NA18558,NA18940,NA18504,NA18501 | 0,6 | 3 kb | High coverage NA12891 (24.5x) |
