# Supplementary material for: Structural variation on the human Y chromosome from population-scale resequencing
Source: Croat Med J. 2015 Jun;56(3):194–207. doi: 10.3325/cmj.2015.56.194 (PMC4500966; doi:10.3325/cmj.2015.56.194)
Supplement: Supplementary Table 3 [file CroatMedJ_56_s003.pdf]

Variation_ID	Chr	Region_Star	Region_End	Outermost_St	Outermost_Er	Type_OF_Ever	Length_(bp)	Max_Length_(	#_Samples	Samples	Population	MSY_Class	GENCODE_Ba	GENCODE_Ba	Main_Discovery_Approac	Main_Data_S	Main_Dataset
CG_SV_11717	Y	13257921	13258340	13257921	13258340	Gains:0,Deleti	419	419	1	NA18504	YRI	Heterochroma	NA	NA	PE.CompleteGenomics	CG	Complete_Genomics_Report
CG_SV_11758	Y	13298067	13298807	13298067	13298807	Gains:0,Deleti	740	740	1	NA12891	CEU	Heterochroma	NA	NA	PE.CompleteGenomics	CG	Complete_Genomics_Report
CG_SV_10578	Y	9968218	9971914	9968220	9971914	Gains:0,Deleti	3696	3694	3	NA06994,NA0	CEU	Other	NA	NA	PE.CompleteGenomics	CG	Complete_Genomics_Report
CG_SV_10578	Y	9968220	9971914	9968220	9971914	Gains:0,Deleti	3694	3694	3	NA12891,NA1	CEU,YRI	Other	NA	NA	PE.CompleteGenomics	CG	Complete_Genomics_Report
CG_SV_10618	Y	10008229	10012623	10008229	10012623	Gains:0,Deleti	4394	4394	1	NA10851	CEU	Other	NA	NA	PE.CompleteGenomics	CG	Complete_Genomics_Report
BD_01	Y	7186834	7187145	7186799	7187161	Gains:0,Deleti	311	362	1	NA06986	CEU	X-Degenerate	PRKY	ENSG0000009	RP.BreakDancer	SLX	This_Work
BD_02	Y	17143551	17143848	17143522	17143873	Gains:0,Deleti	297	351	1	NA11829	CEU	X-Degenerate	NA	NA	RP.BreakDancer	SLX	This_Work
BD_03	Y	17936753	17937022	17936737	17937037	Gains:0,Deleti	269	300	1	NA11919	CEU	X-Degenerate	NA	NA	RP.BreakDancer	SLX	This_Work
BD_04	Y	17149972	17150263	17149957	17150290	Gains:0,Deleti	291	333	1	NA12154	CEU	X-Degenerate	NA	NA	RP.BreakDancer	SLX	This_Work
BD_05	Y	17401807	17402065	17401763	17402093	Gains:0,Deleti	258	330	1	NA18853	YRI	X-Degenerate	NA	NA	RP.BreakDancer	SLX	This_Work

Variation_ID	Linked_Redu	Comment	Y-Chromoso	Pilot_1_DOC_	Pilot_1_PE_T	Complete_Ge	Complete_Ge	Pilot_1_Repo	Phase_1_Rep	OMNI_This_	Literature	PCR	#_OMNI_SNP	#_Sources_Of	Sources_Of_V	Comment_Val	Dataset_Statu	Validation_Status
CG_SV_11717	NA	Repeats	No	-	-	-	+	-	-	-	-	-	0	1	NA	Failed_PCR	Non-Redunda	Unvalidated
CG_SV_11758	NA	Repeats	No	-	-	-	+	-	-	-	-	-	0	1	NA	Failed_PCR	Non-Redunda	Unvalidated
CG_SV_10578	NA	Repeats	No	-	-	-	+	-	-	-	-	-	0	1	NA	Failed_PCR	Non-Redunda	Unvalidated
CG_SV_10578	NA	Repeats	No	-	-	-	+	-	-	-	-	-	0	1	NA	Failed_PCR	Non-Redunda	Unvalidated
CG_SV_10618	NA	Repeats	No	-	-	-	+	-	-	-	-	-	0	1	NA	Failed_PCR	Non-Redunda	Unvalidated
BD_01	NA	BreakDancer_Call	No	-	+	-	-	-	-	-	-	-	0	1	NA	Failed_PCR	Non-Redunda	Unvalidated
BD_02	NA	BreakDancer_Call	No	-	+	-	-	-	-	-	-	-	0	1	NA	Failed_PCR	Non-Redunda	Unvalidated
BD_03	NA	BreakDancer_Call	No	-	+	-	-	-	-	-	-	-	0	1	NA	Failed_PCR	Non-Redunda	Unvalidated
BD_04	NA	BreakDancer_Call	No	-	+	-	-	-	-	-	-	-	0	1	NA	Failed_PCR	Non-Redunda	Unvalidated
BD_05	NA	BreakDancer_Call	No	-	+	-	-	-	-	-	-	-	0	1	NA	Failed_PCR	Non-Redunda	Unvalidated
