# Supplementary material for: Structural variation on the human Y chromosome from population-scale resequencing
Source: Croat Med J. 2015 Jun;56(3):194–207. doi: 10.3325/cmj.2015.56.194 (PMC4500966; doi:10.3325/cmj.2015.56.194)
Supplement: Supplementary Table 4 [file CroatMedJ_56_s004.pdf]

Variation_ID	Chr	Region_Start	Region_End	Outermost_St	Outermost_Er	Local_Fragme	Local_Fragme	Type_OF_Ever	Length_(bp)	Max_Length_(	#_Samples	Samples	Population
CG_SV_11717921_11718340	Y	13257921	13258340	13257921	13258340	13257921	13258340	Del	419	419	1	NA18504	YRI
CG_SV_11758067_11758807	Y	13298067	13298807	13298067	13298807	13298067	13298807	Del	740	740	1	NA12891	CEU
CG_SV_10578218_10581914	Y	9968218	9971914	9968220	9971914	9968218	9971914	Del	3696	3694	6	NA06994	CEU
CG_SV_10578220_10581914	Y	9968220	9971914	9968220	9971914	9968220	9971914	Del	3694	3694	6	NA07357	CEU
CG_SV_10578220_10581914	Y	9968220	9971914	9968220	9971914	9968220	9971914	Del	3694	3694	6	NA10851	CEU
CG_SV_10578220_10581914	Y	9968220	9971914	9968220	9971914	9968220	9971914	Del	3694	3694	6	NA12891	CEU
CG_SV_10578218_10581914	Y	9968218	9971914	9968220	9971914	9968218	9971914	Del	3696	3694	6	NA18501	YRI
CG_SV_10578218_10581914	Y	9968218	9971914	9968220	9971914	9968218	9971914	Del	3696	3694	6	NA18504	YRI
CG_SV_10618229_10622623	Y	10008229	10012623	10008229	10012623	10008229	10012623	Del	4394	4394	1	NA10851	CEU
BD_01	Y	7186834	7187145	7186799	7187161	7186834	7187145	Del	311	362	1	NA06986	CEU
BD_02	Y	17143551	17143848	17143522	17143873	17143551	17143848	Del	297	351	1	NA11829	CEU
BD_03	Y	17936753	17937022	17936737	17937037	17936753	17937022	Del	269	300	1	NA11919	CEU
BD_04	Y	17149972	17150263	17149957	17150290	17149972	17150263	Del	291	333	1	NA12154	CEU
BD_05	Y	17401807	17402065	17401763	17402093	17401807	17402065	Del	258	330	1	NA18853	YRI

Variation_ID	MSY_Class	SegDup_Regi	GENCODE_Ba	GENCODE_Ba	Main_Discove	Main_Data_S	Main_Dataset	Linked_Redun	1000_Genom	Comment	Y-Chromosom	Pilot_1_DOC_	Pilot_1_PE_T
CG_SV_11717921_11718340	Heterochroma	chr1:1420800	NA	NA	PE.CompleteG	CG	Complete_Ger	NA	Pilot_1	Repeats	No	-	-
CG_SV_11758067_11758807	Heterochroma	chr1:1420800	NA	NA	PE.CompleteG	CG	Complete_Ger	NA	Pilot_1	Repeats	No	-	-
CG_SV_10578218_10581914	Other	chr5:4027249	NA	NA	PE.CompleteG	CG	Complete_Ger	NA	Pilot_1	Repeats	No	-	-
CG_SV_10578220_10581914	Other	chr5:4027249	NA	NA	PE.CompleteG	CG	Complete_Ger	NA	Pilot_1	Repeats	No	-	-
CG_SV_10578220_10581914	Other	chr5:4027249	NA	NA	PE.CompleteG	CG	Complete_Ger	NA	Pilot_1	Repeats	No	-	-
CG_SV_10578220_10581914	Other	chr5:4027249	NA	NA	PE.CompleteG	CG	Complete_Ger	NA	Pilot_1	Repeats	No	-	-
CG_SV_10578218_10581914	Other	chr5:4027249	NA	NA	PE.CompleteG	CG	Complete_Ger	NA	Pilot_1	Repeats	No	-	-
CG_SV_10578218_10581914	Other	chr5:4027249	NA	NA	PE.CompleteG	CG	Complete_Ger	NA	Pilot_1	Repeats	No	-	-
CG_SV_10618229_10622623	Other	chr16:338291	NA	NA	PE.CompleteG	CG	Complete_Ger	NA	Pilot_1	Repeats	No	-	-
BD_01	X-Degenerate	chrX:3582997	PRKY	ENSG0000009	RP.BreakDanc	SLX	This_Work	NA	Pilot_1	BreakDancer_	No	-	+
BD_02	X-Degenerate	NA	NA	NA	RP.BreakDanc	SLX	This_Work	NA	Pilot_1	BreakDancer_	No	-	+
BD_03	X-Degenerate	NA	NA	NA	RP.BreakDanc	SLX	This_Work	NA	Pilot_1	BreakDancer_	No	-	+
BD_04	X-Degenerate	NA	NA	NA	RP.BreakDanc	SLX	This_Work	NA	Pilot_1	BreakDancer_	No	-	+
BD_05	X-Degenerate	NA	NA	NA	RP.BreakDanc	SLX	This_Work	NA	Pilot_1	BreakDancer	No	-	+

Variation_ID	Complete_Ge	Complete_Ge	Pilot_1_Repo	Phase_1_Rep	OMNI_This_	Literature	PCR	#_OMNI_SNP	#_Sets_Overl	Sources_Of_Val	Comment_Validatio	Dataset_Statu	Validation_Status
CG_SV_11717921_11718340	-	+	-	-	-	-	-	0	1	NA	Failed_PCR	Non-Redunda	Unvalidated
CG_SV_11758067_11758807	-	+	-	-	-	-	-	0	1	NA	Failed_PCR	Non-Redunda	Unvalidated
CG_SV_10578218_10581914	-	+	-	-	-	-	-	0	1	NA	Failed_PCR	Non-Redunda	Unvalidated
CG_SV_10578220_10581914	-	+	-	-	-	-	-	0	1	NA	Failed_PCR	Non-Redunda	Unvalidated
CG_SV_10578220_10581914	-	+	-	-	-	-	-	0	1	NA	Failed_PCR	Non-Redunda	Unvalidated
CG_SV_10578220_10581914	-	+	-	-	-	-	-	0	1	NA	Failed_PCR	Non-Redunda	Unvalidated
CG_SV_10578218_10581914	-	+	-	-	-	-	-	0	1	NA	Failed_PCR	Non-Redunda	Unvalidated
CG_SV_10578218_10581914	-	+	-	-	-	-	-	0	1	NA	Failed_PCR	Non-Redunda	Unvalidated
CG_SV_10618229_10622623	-	+	-	-	-	-	-	0	1	NA	Failed_PCR	Non-Redunda	Unvalidated
BD_01	-	-	-	-	-	-	-	0	1	NA	Failed_PCR	Non-Redunda	Unvalidated
BD_02	-	-	-	-	-	-	-	0	1	NA	Failed_PCR	Non-Redunda	Unvalidated
BD_03	-	-	-	-	-	-	-	0	1	NA	Failed_PCR	Non-Redunda	Unvalidated
BD_04	-	-	-	-	-	-	-	0	1	NA	Failed_PCR	Non-Redunda	Unvalidated
BD_05	-	-	-	-	-	-	-	0	1	NA	Failed_PCR	Non-Redunda	Unvalidated
