# Supplementary material for: Structural variation on the human Y chromosome from population-scale resequencing
Source: Croat Med J. 2015 Jun;56(3):194–207. doi: 10.3325/cmj.2015.56.194 (PMC4500966; doi:10.3325/cmj.2015.56.194)

Supplementary Material

Graphic Support for SV Events

Description

For each of the SV regions, a selection of the best graphic supporting material is shown when available. All evidence presented here was produced as part of this study.

[P1T] Pilot 1 This Work. Evidence from a depth of coverage approach using Pilot 1 (Illumina) low-coverage samples from the 1000 Genomes Project.

[P1T] Pilot 1 This Work - Haplogroup Analysis. Evidence from a depth of coverage approach using medium-coverage merged samples representative of each Y haplogroup.

[CGT] Complete Genomics This Work. Evidence from a depth of coverage approach using high-coverage samples from Complete Genomics public repository.

[OCT] OMNI Chip This Work. Evidence from 2.5 OMNI intensity arrays over Pilot 1 samples from the 1000 Genomes Project. When present, supporting figures are always displayed in pairs. Figures labeled as «event», show evidence either for a deletion or gain. Figures labeled as «no event», serve as a reference (usually NA10851) of how a sample with no deletion/gain looks like.

[CGR] Complete Genomics Report. No graphic evidence available.

[P1R] Pilot 1 Report. No graphic evidence available.

[Ph1R] Phase 1 Report. No graphic evidence available.

[L] Literature Evidence. No graphic evidence available.

[PCR] PCR This Work. Evidence from PCR analysis.

[Segmental Duplications]. Custom track that shows segmental duplications within and around SVs.

Red Lines (vertical). They are positioned at the start and end limits of SV boundaries.

Blue Lines (horizintal). They are positioned at log2 ratios -0.6 and 0.6. They serve as a reference of standard minimum thresholds for a deletion and gain (respectively). This study do not strictly follows this criterion.

Haplogroups

Haplogroups<br>(as they appear in the figures)	Haplogroups<br>(as they must be read in the figures)
N	N
C	C
O3e	O3a
R1b1b2	R1b1a2a1a2b
O2b	O2b
D	D1b
R1	R1
E1b1a	E1b1a1a1
I1	I1
E1b1a8a	E1b1a1a1d1
O	O

Region SV 01
NA
Evidence: Ph1R + PCR
From 3,109,266 To 3,111,300
Length: 2,034 bp
Present in 4 samples

PCR

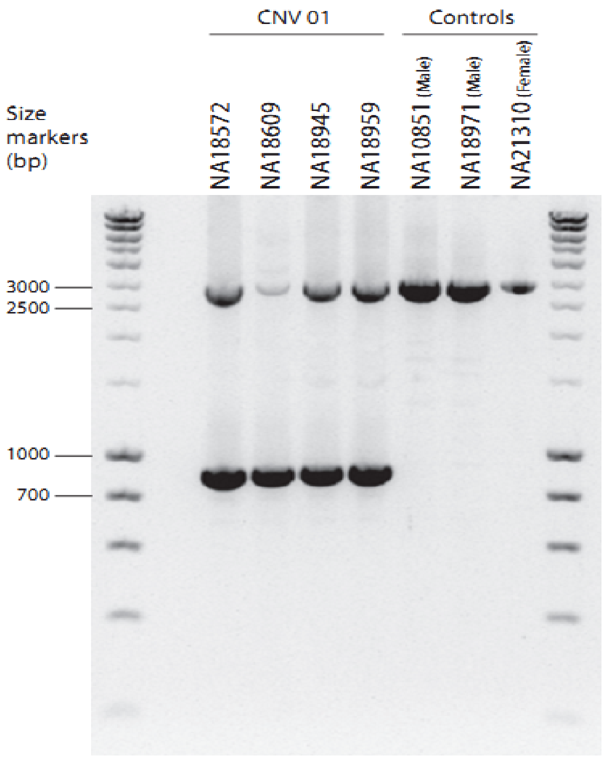

Segmental Duplications

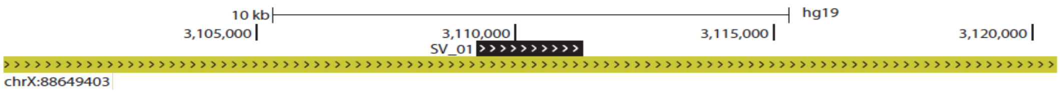

Region SV 02
New
Evidence: P1T + CGT
From 6,543,750 To 6,573,750
Length: 30,000 bp
Present in 22 samples

Pilot 1 This Work NA18561_CHB_O2b

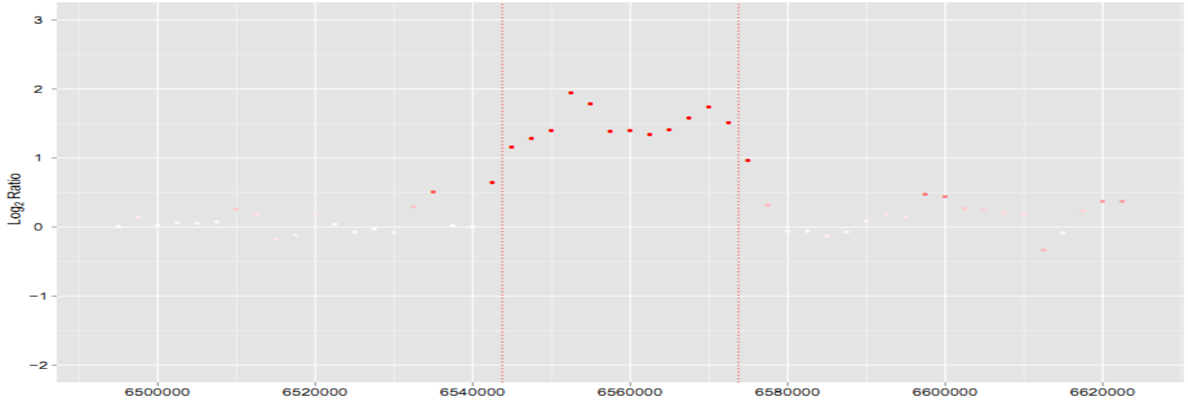

Pilot 1 This Work - Haplogroup Analysis Haplogroup_R1

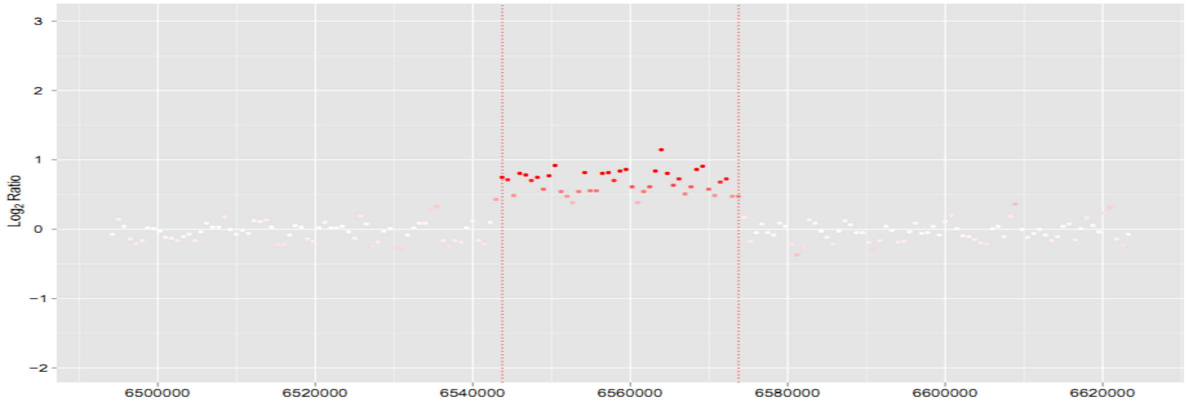

Complete Genomics This Work NA18940_JPT_D

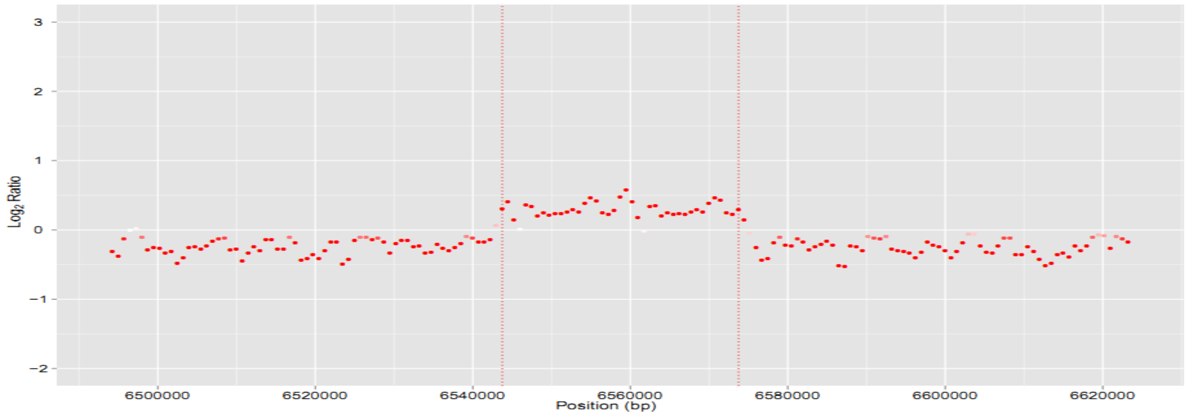

Segmental Duplications

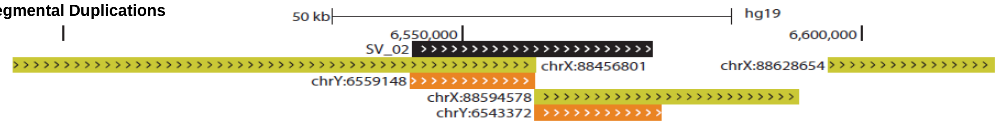

Region SV 03
New
Evidence: P1T + OCT
From 7,761,250 To 8,001,250
Length: 240,000 bp
Present in 1 sample

Pilot 1 This Work

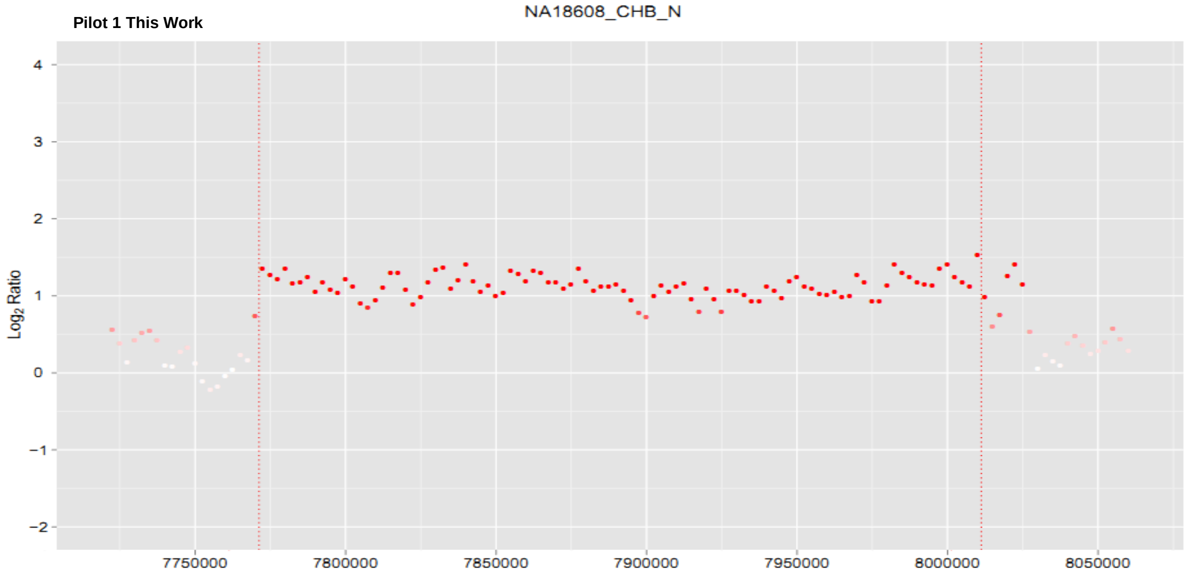

Segmental Duplications

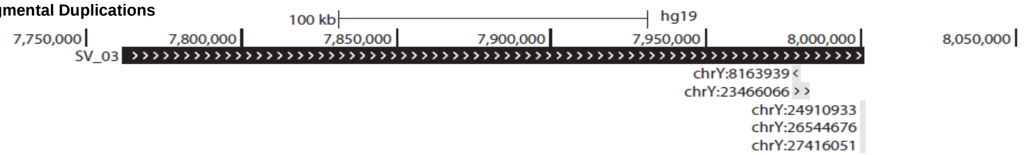

OMNI Chip This Work - Event

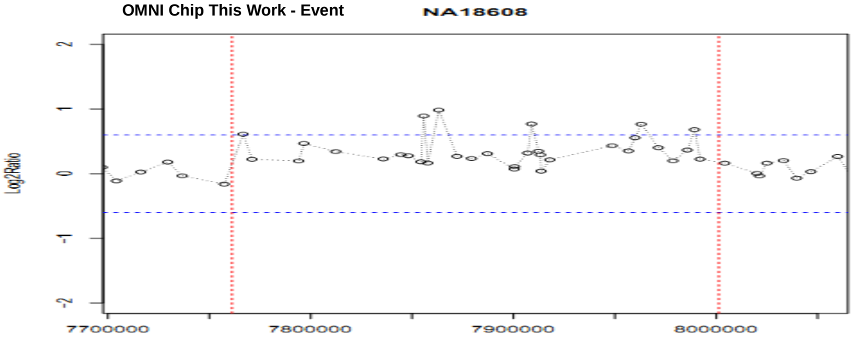

OMNI Chip This Work - No Event

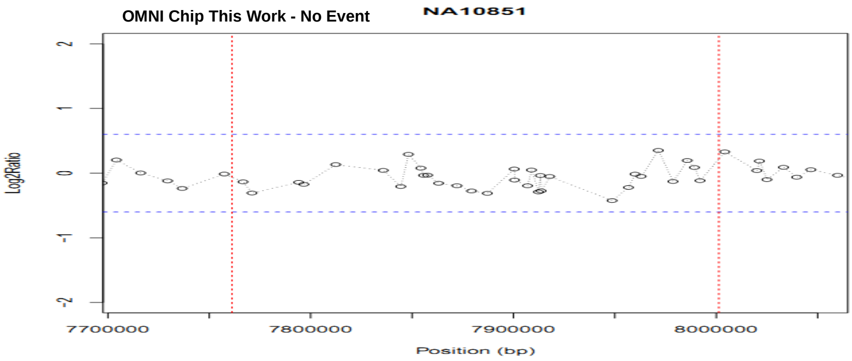

Region SV 04
TSPY array
Evidence: P1T + CGT + OCT + L
From 9,172,875 To 9,236,625
Length: 63,750 bp
Present in 5 samples

Pilot 1 This Work - Haplogroup Analysis

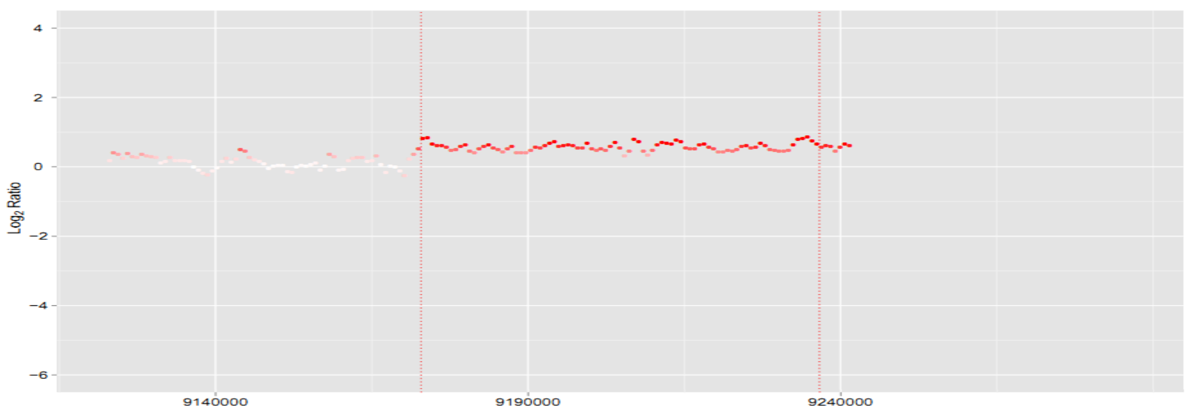

Complete Genomics This Work

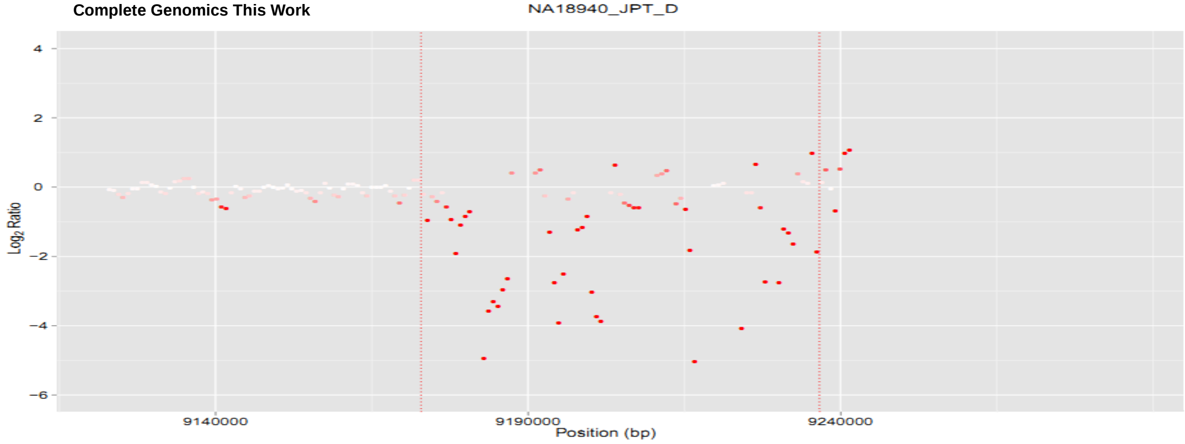

Segmental Duplications

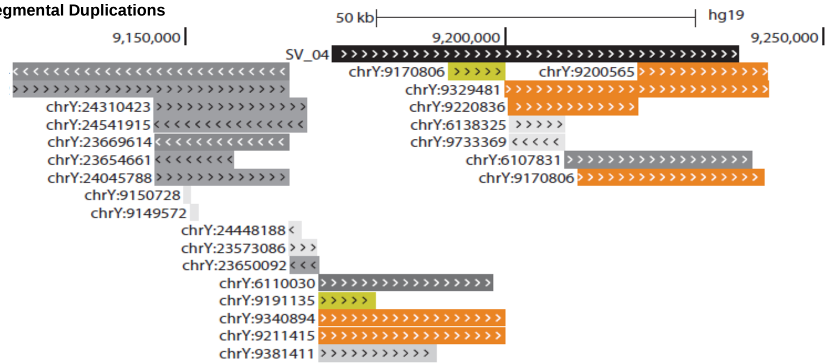

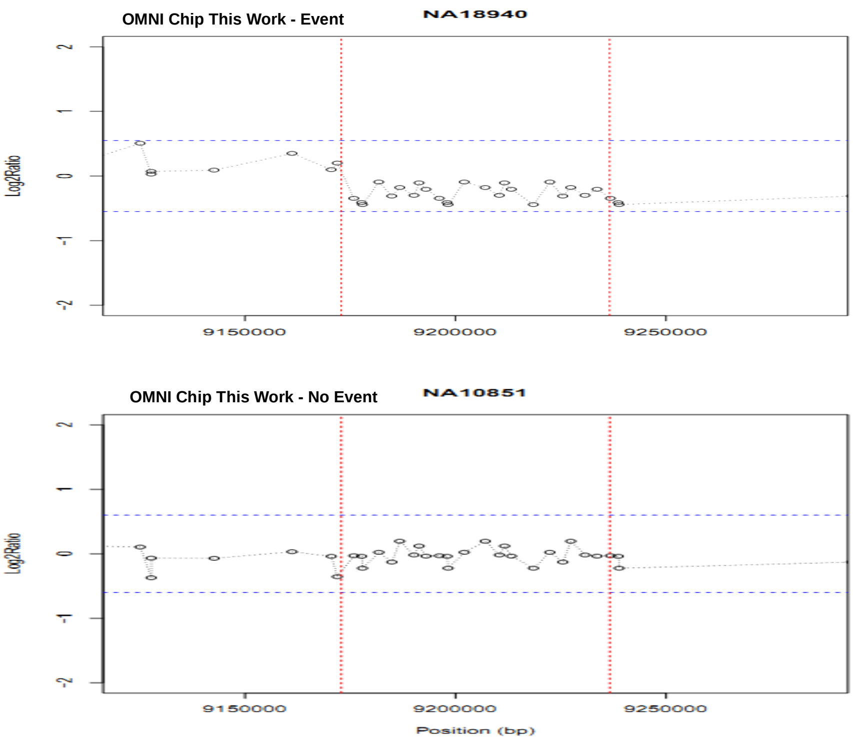
OMNI Chip This Work - Event
NA18940
Log2Ratio
2
1
0
-1
-2
9150000
9200000
9250000
OMNI Chip This Work - No Event
NA10851
Log2Ratio
2
1
0
-1
-2
9150000
9200000
9250000
Position (bp)

Region SV 05
TSPY array
Evidence: P1T + OCT + L
From 9,300,125 To 9,367,625
Length: 67,500 bp
Present in 6 samples

Pilot 1 This Wok

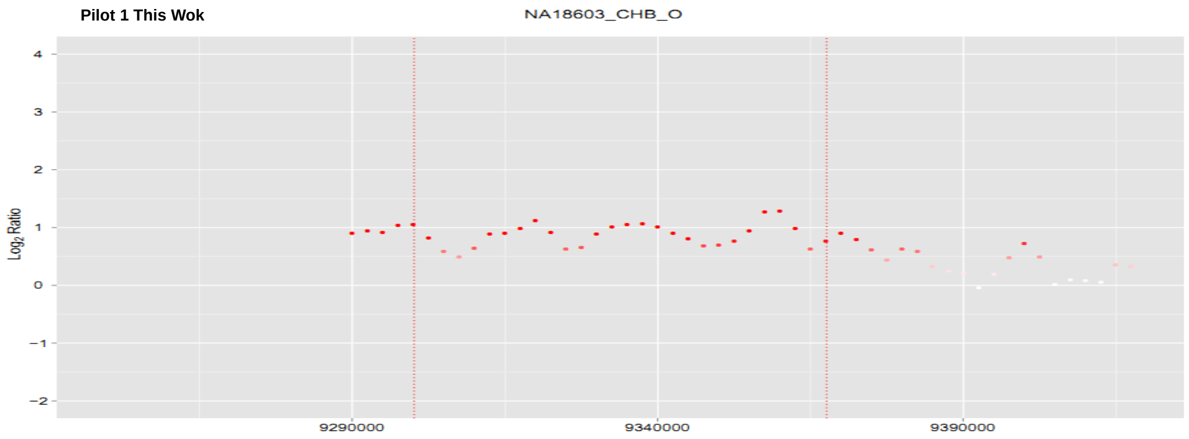

Pilot 1 This Work - Haplogroup Analysis

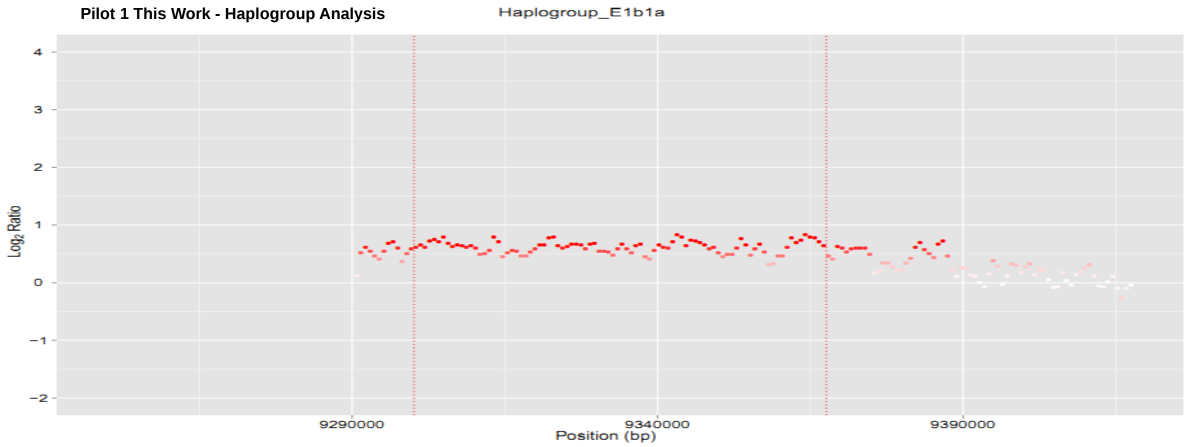

Segmental Duplications

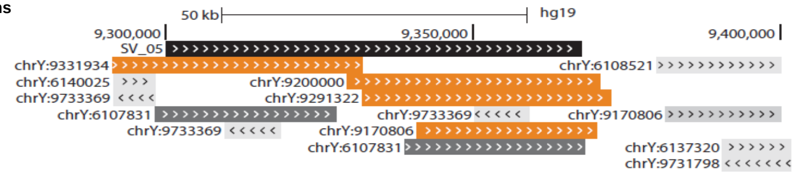

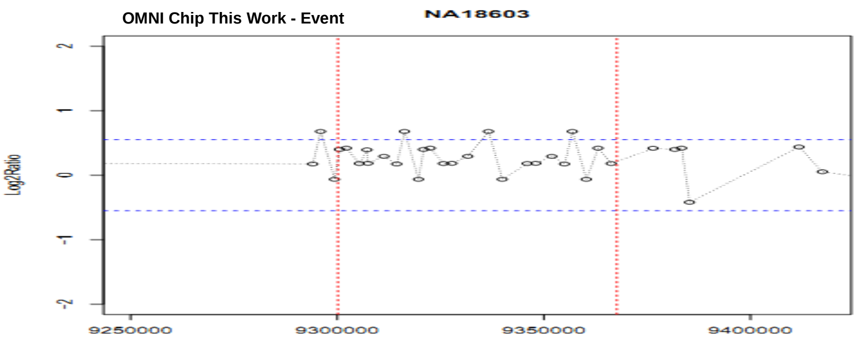
OMNI Chip This Work - Event
NA18603
Log2Ratio
2
1
0
-1
-2
9250000
9300000
9350000
9400000

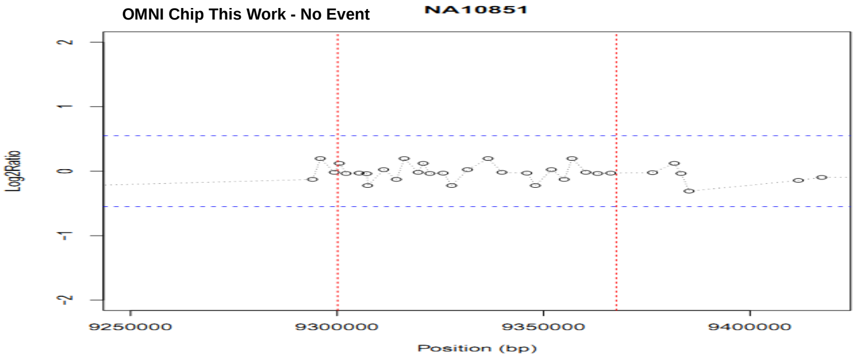
OMNI Chip This Work - No Event
NA10851
Log2Ratio
2
1
0
-1
-2
9250000
9300000
9350000
9400000
Position (bp)

Region SV 06
TTTY22
Evidence: P1T + CGT + CGR + OCT
From 9,639,875 To 9,650,375
Length: 10,500 bp
Present in 8 samples

Pilot 1 This Work NA18501_YRI_E1b1a8a

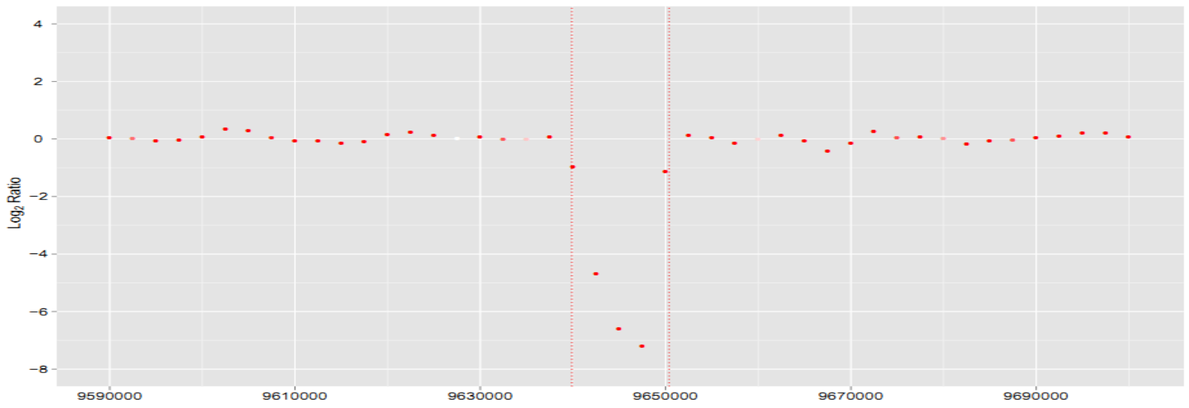

Pilot 1 This Work - Haplogroup Analysis Haplogroup_E1b1a

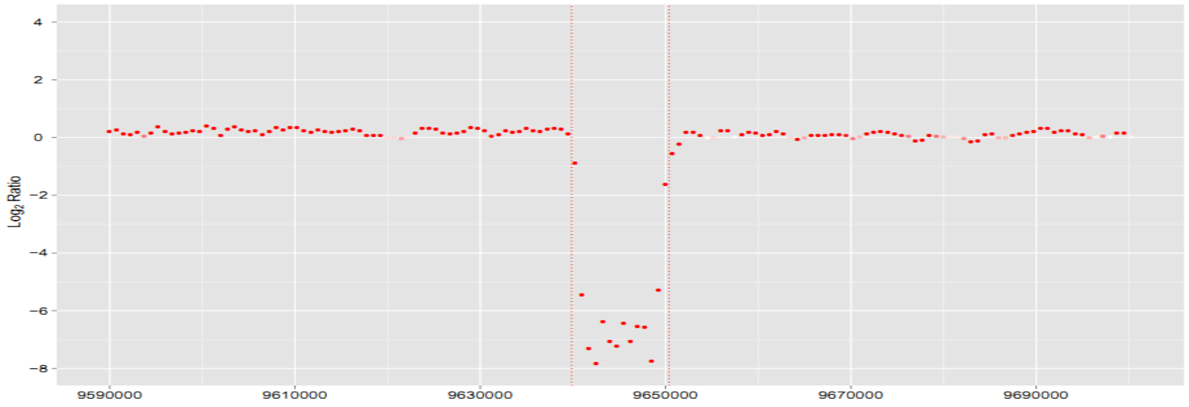

Complete Genomics This Work NA18501_YRI_E1b1a8a

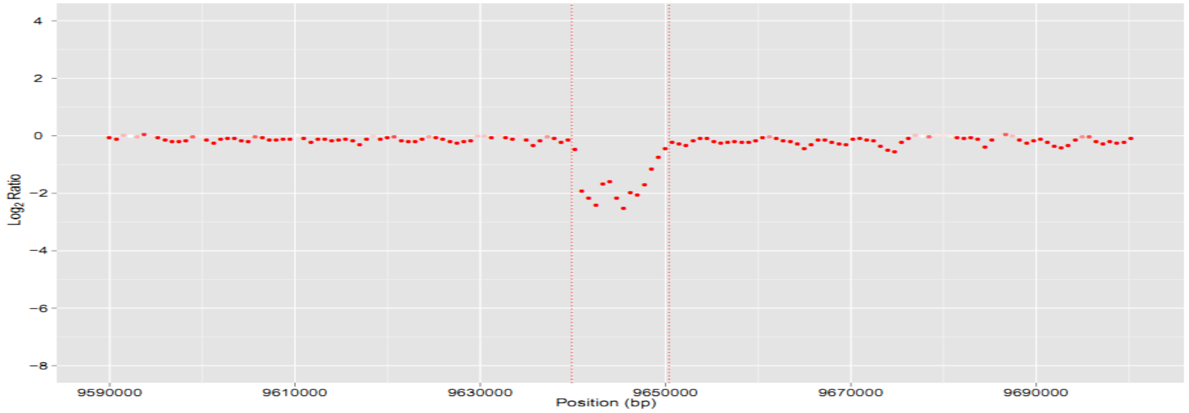

Segmental Duplications

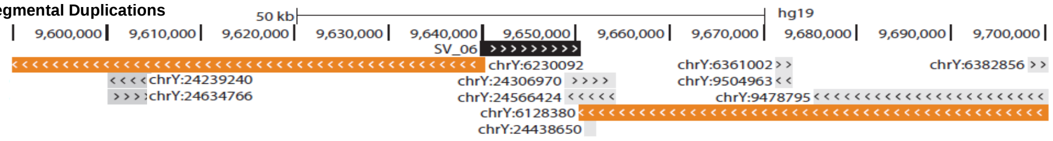

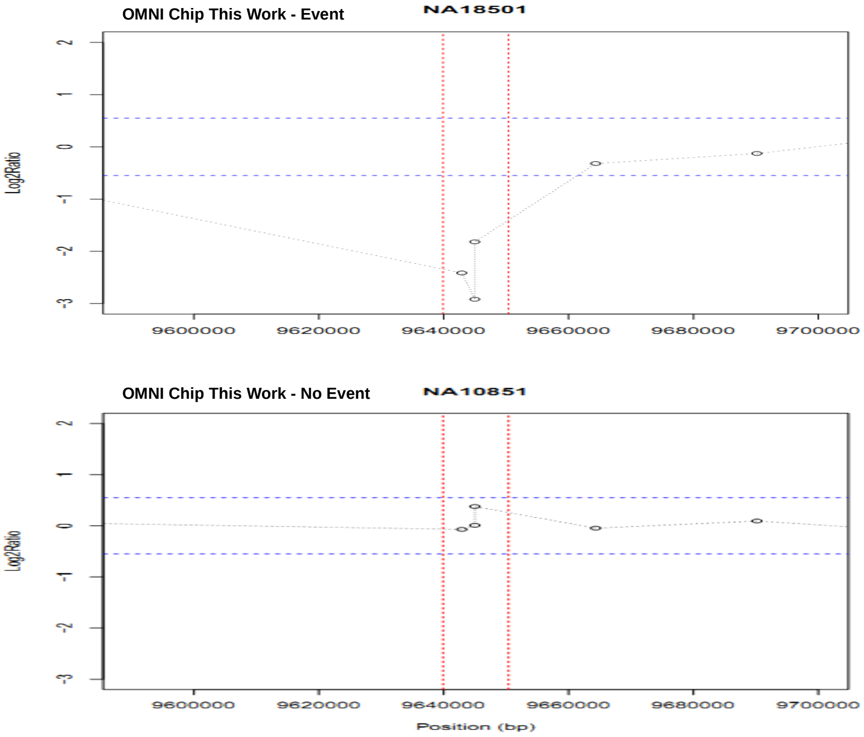
OMNI Chip This Work - Event
NA18501
Log2Ratio
2
1
0
-1
-2
-3
9600000
9620000
9640000
9660000
9680000
9700000
OMNI Chip This Work - No Event
NA10851
Log2Ratio
2
1
0
-1
-2
-3
9600000
9620000
9640000
9660000
9680000
9700000
Position (bp)

Region SV 07
Alphoid Repeats
Evidence: P1T + L
From 10,016,250 To 10,041,250
Length: 25,000 bp
Present in 37 samples

Pilot 1 This Work

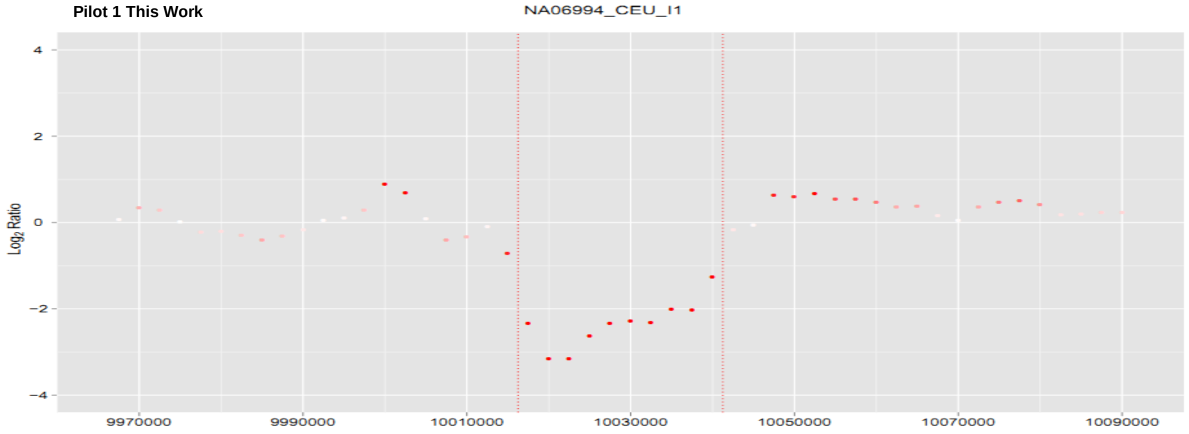

Pilot 1 This Work - Haplogroup Analysis

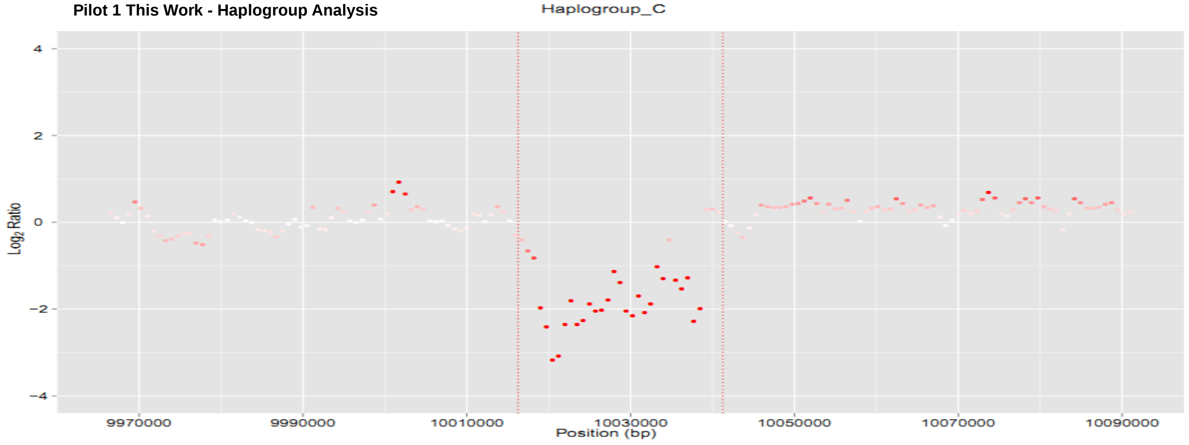

Segmental Duplications

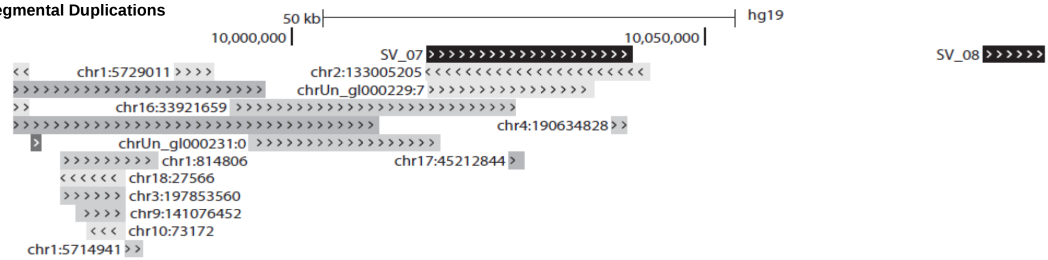

Region SV 08
Alphoid Repeats
Evidence: P1T + CGT + OCT + L
From 10,083,750 To 10,104,553
Length: 20,803 bp
Present in 13 samples

Pilot 1 This Work

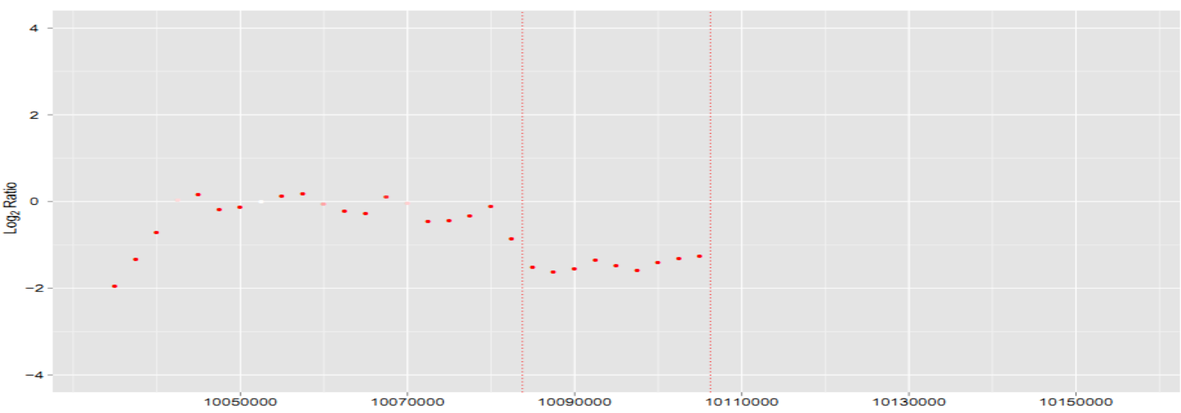

Pilot 1 This Work - Haplogroup Analysis

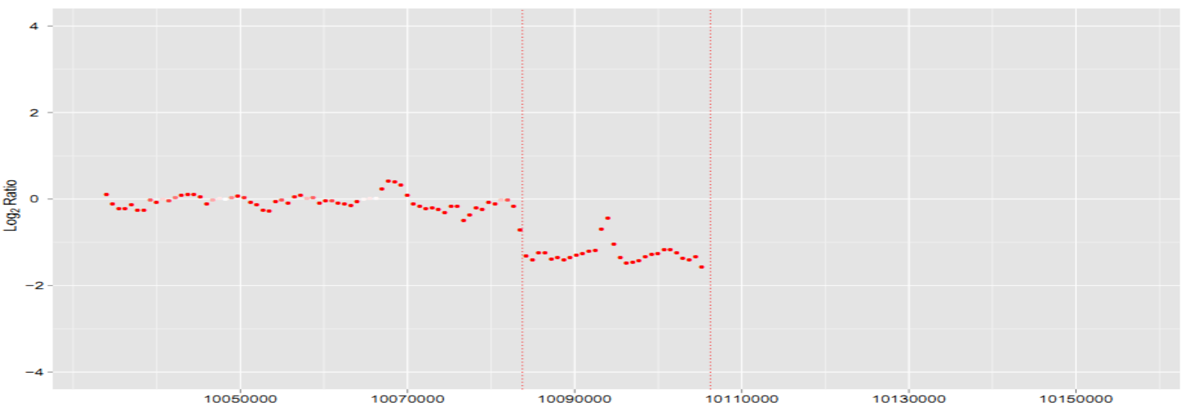

Complete Genomics This Work

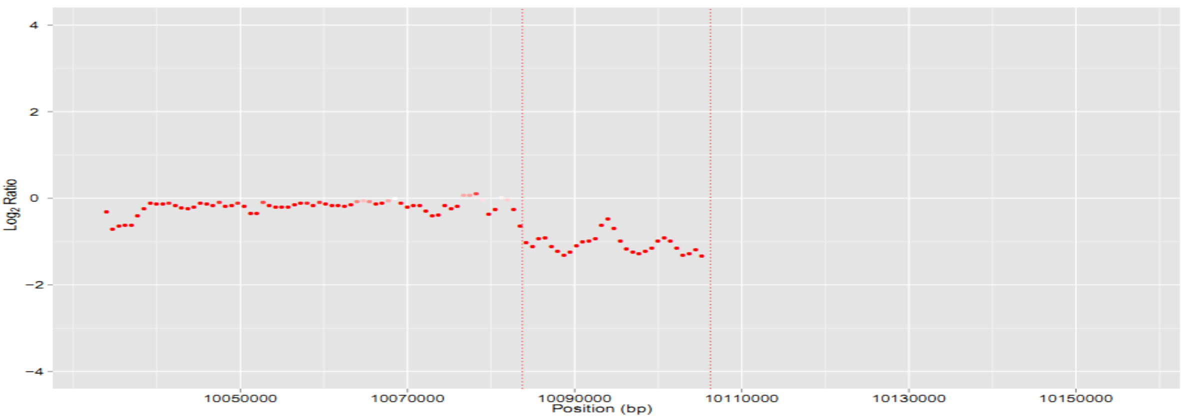

Segmental Duplications

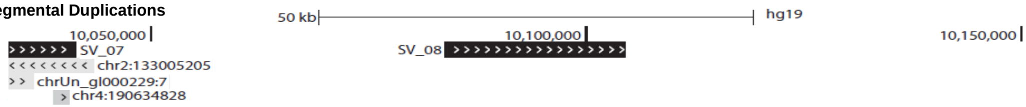

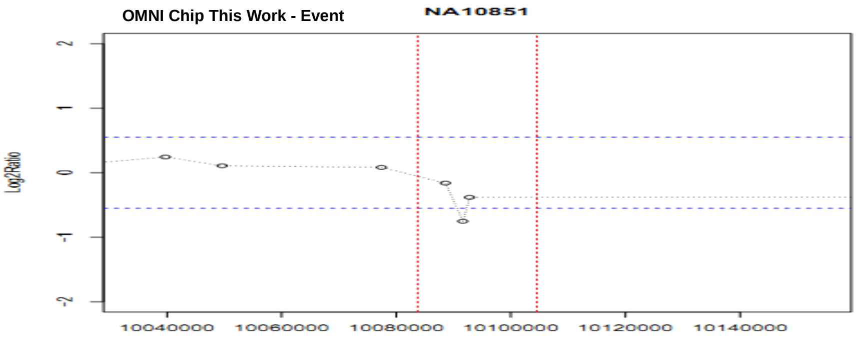

OMNI Chip This Work - Event
NA10851
Log2Ratio
2
1
0
-1
-2
10040000
10060000
10080000
10100000
10120000
10140000

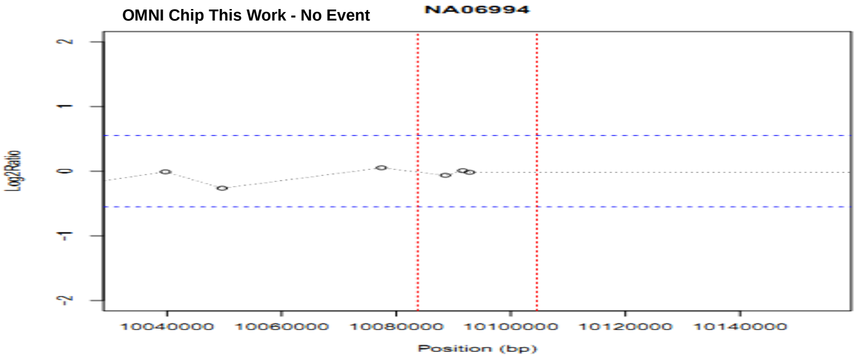

OMNI Chip This Work - No Event
NA06994
Log2Ratio
2
1
0
-1
-2
10040000
10060000
10080000
10100000
10120000
10140000
Position (bp)

Region SV 09
Repeats
Evidence: P1T + CGT + OCT + L
From 13,104,553 To 1 3,126,250
Length: 21,697 bp
Present in 17 samples

Pilot 1 This Work

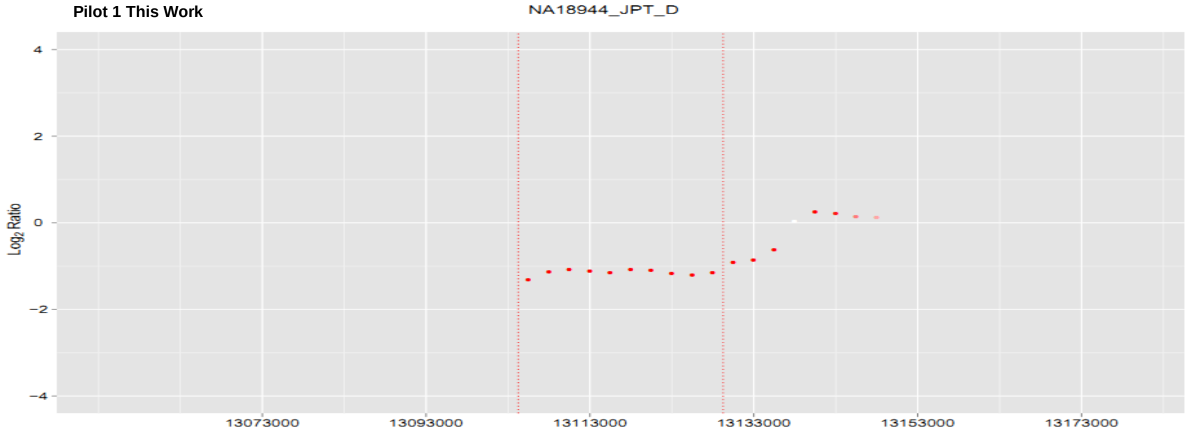

Pilot 1 This Work - Haplogroup Analysis

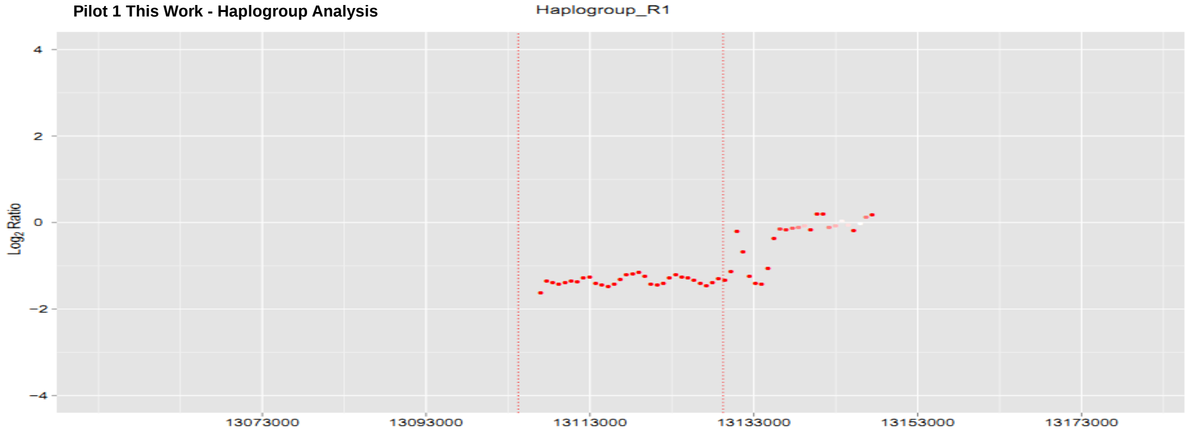

Complete Genomics This Work

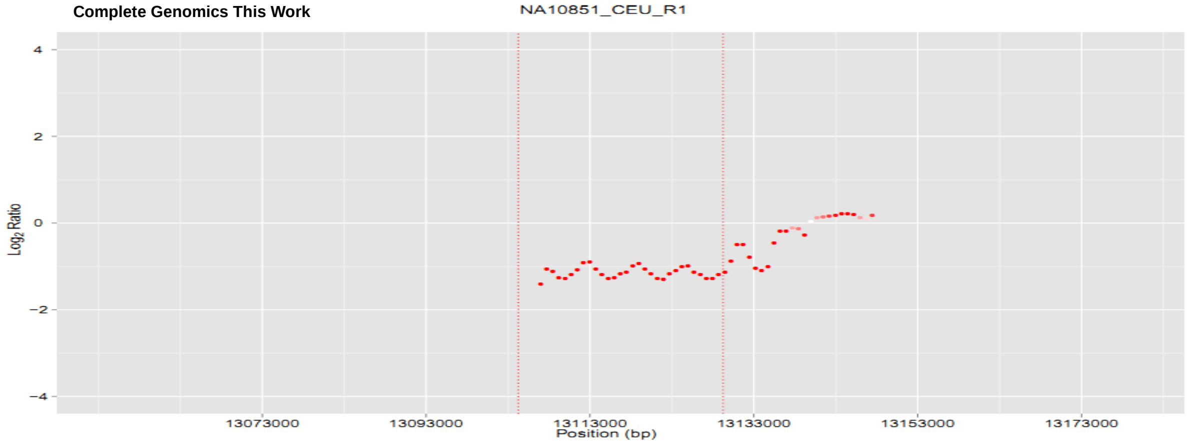

Segmental Duplications

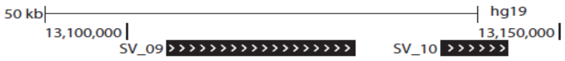

OMNI Chip This Work - Event

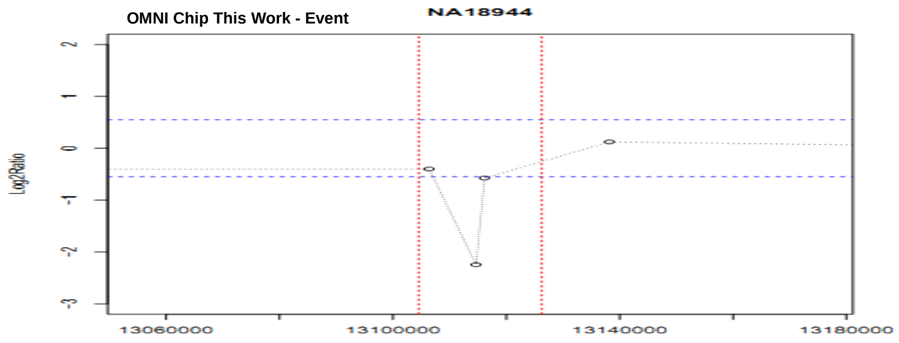

OMNI Chip This Work - No Event

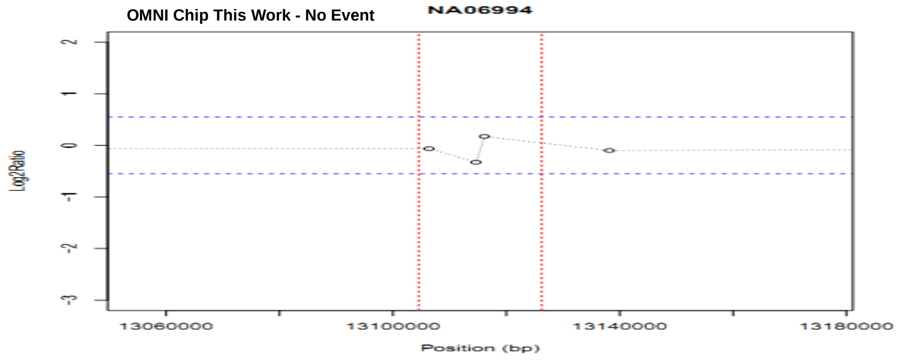

Region SV 10
Repeats
Evidence: P1T + L
From 13,136,250 To 13,143,954
Length: 7,704 bp
Present in 9 samples

Pilot 1 This Work

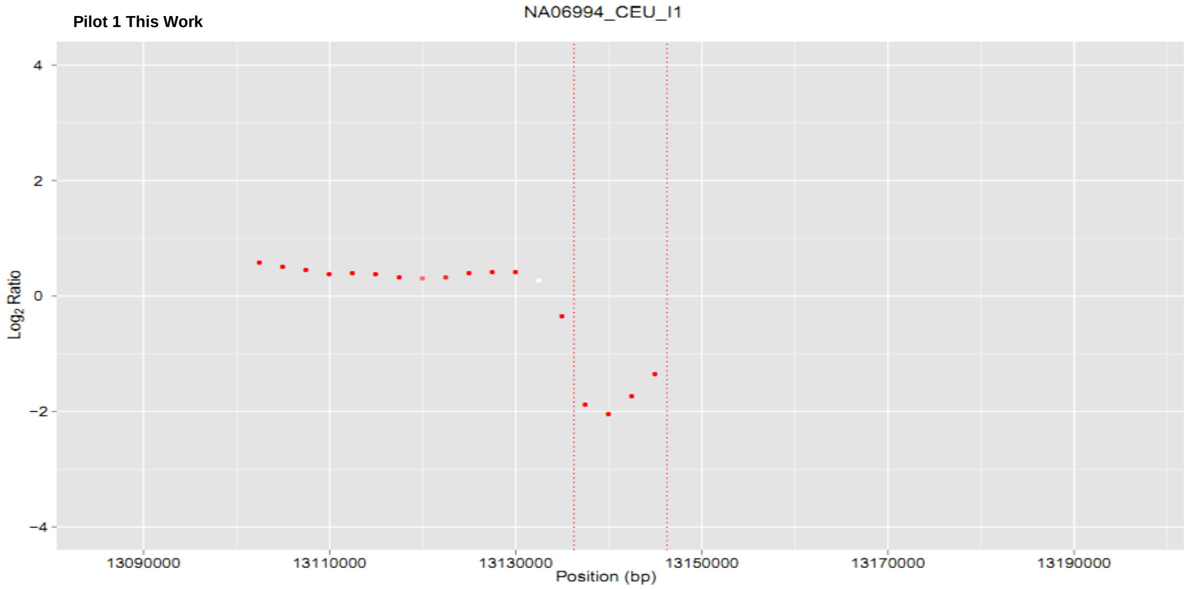

Segmental Duplications

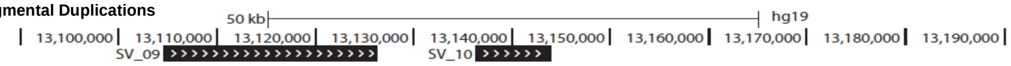

Region SV 11
Repeats
Evidence: P1T + CGR + L
From 13,446,250 To 13,688,750
Length: 242,500 bp
Present in 40 samples

Pilot 1 This Work

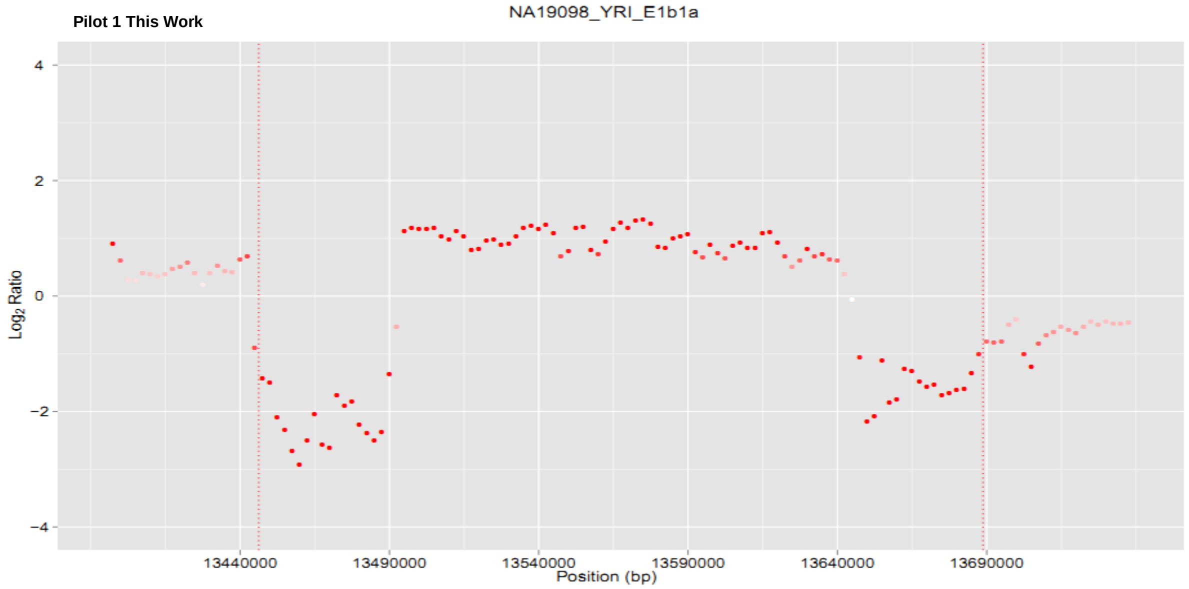

Segmental Duplications

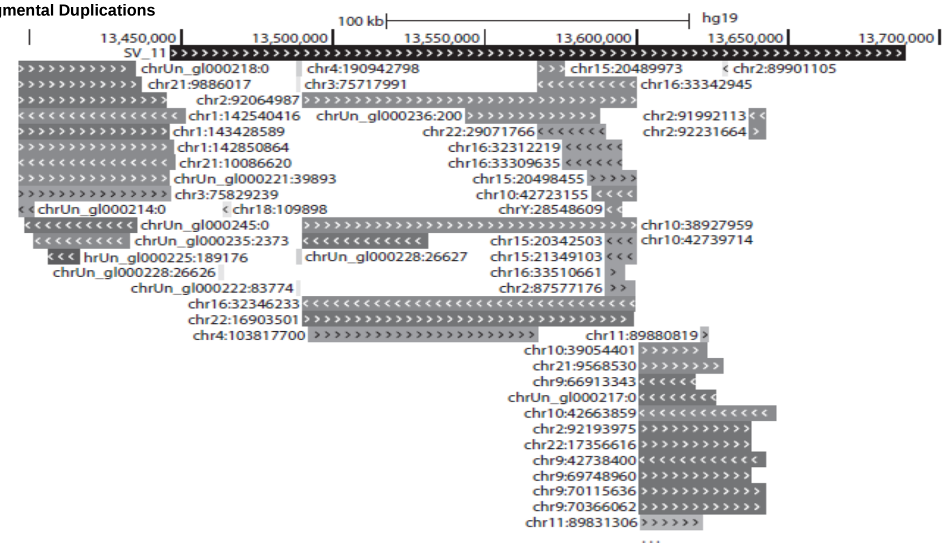

Region SV 12
NA
Evidence: P1R + PCR
From 14,208,831 To 14,208,912
Length: 81bp
Present in 4 samples

No Graphic Support Available

Segmental Duplications

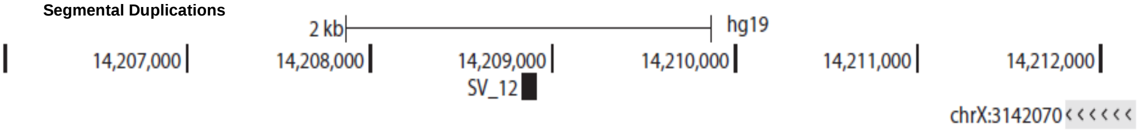

Region SV 13
NA
Evidence: P1T + Ph1R + OCT + PCR
From 17,306,559 To 17,311,584
Length: 5,025 bp
Present in 3 samples

Pilot 1 This Work

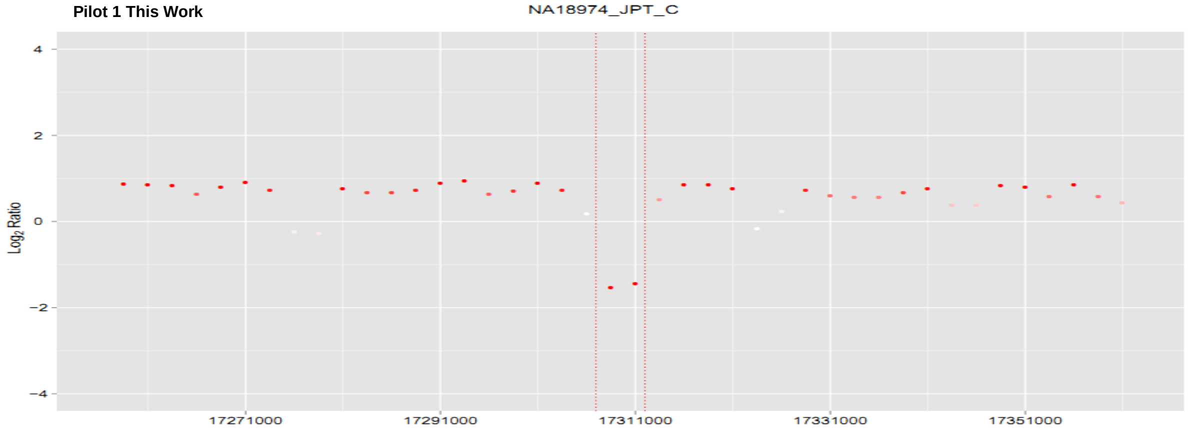

Pilot 1 This Work - Haplogroup Analysis

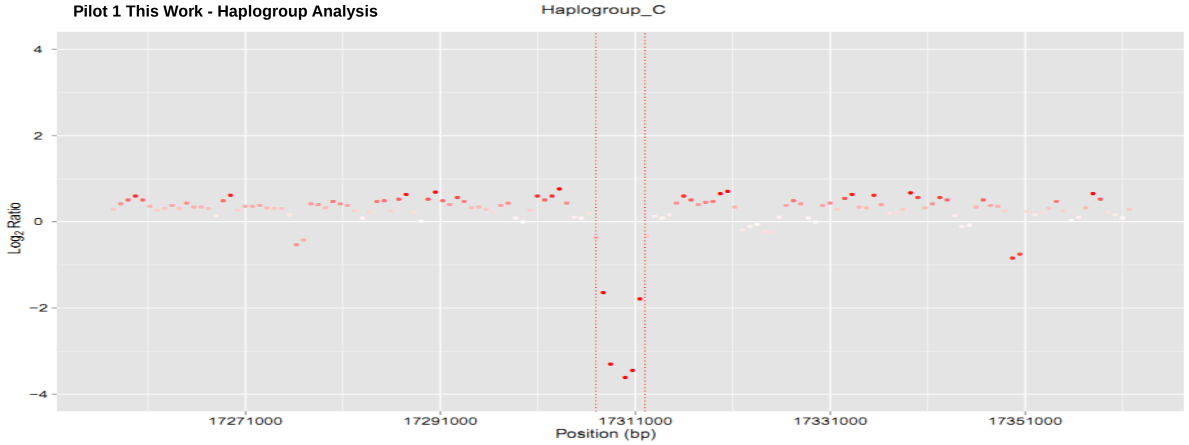

Segmental Duplications

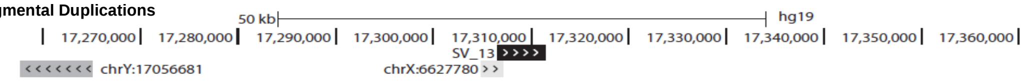

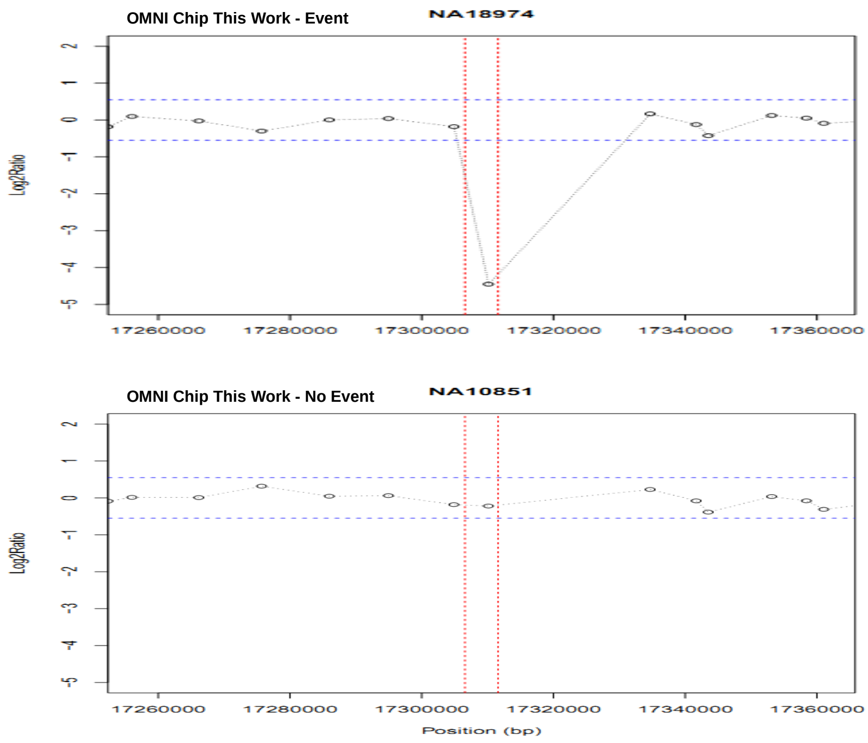
OMNI Chip This Work - Event
NA18974
Log2Ratio
2
1
0
-1
-2
-3
-4
-5
17260000
17280000
17300000
17320000
17340000
17360000
OMNI Chip This Work - No Event
NA10851
Log2Ratio
2
1
0
-1
-2
-3
-4
-5
17260000
17280000
17300000
17320000
17340000
17360000
Position (bp)

PCR

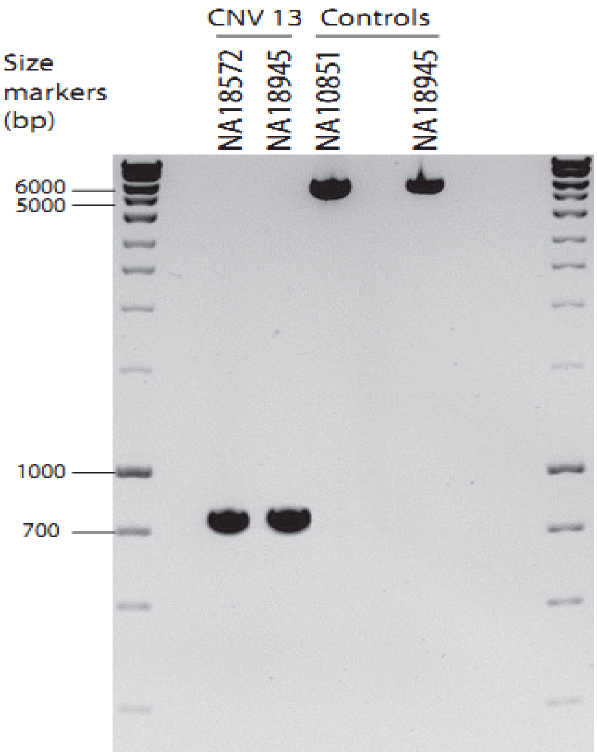

CNV 13
Controls
Size markers (bp)
NA18572
NA18945
NA10851
NA18945
6000
5000
1000
700

Region SV 14
DYZ19
Evidence: P1T + CGT + CGR + Ph1R
From 22,223,737 To 22,434,987
Length: 211,250 bp
Present in 64 samples

Pilot 1 This Work

NA18853_YRI_E1b1a8a

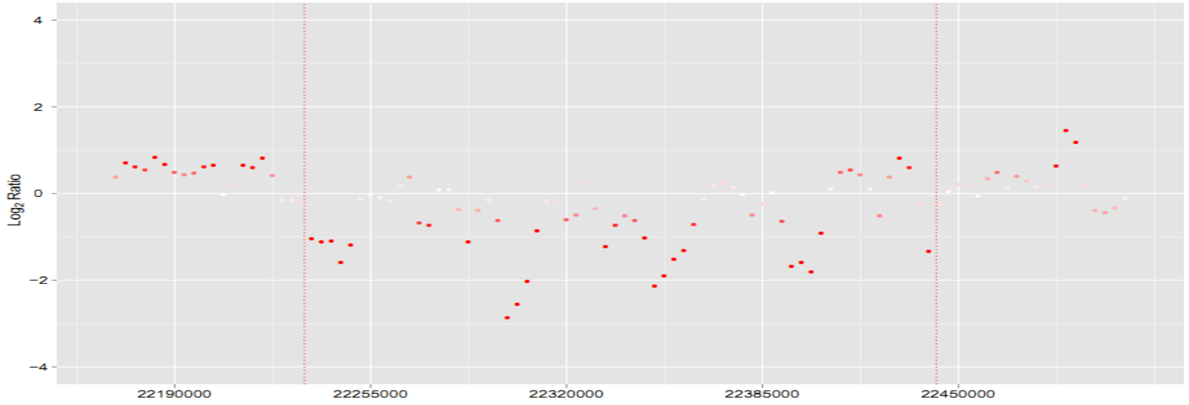

Pilot 1 This Work - Haplogroup Analysis

Haplogroup_E1b1a

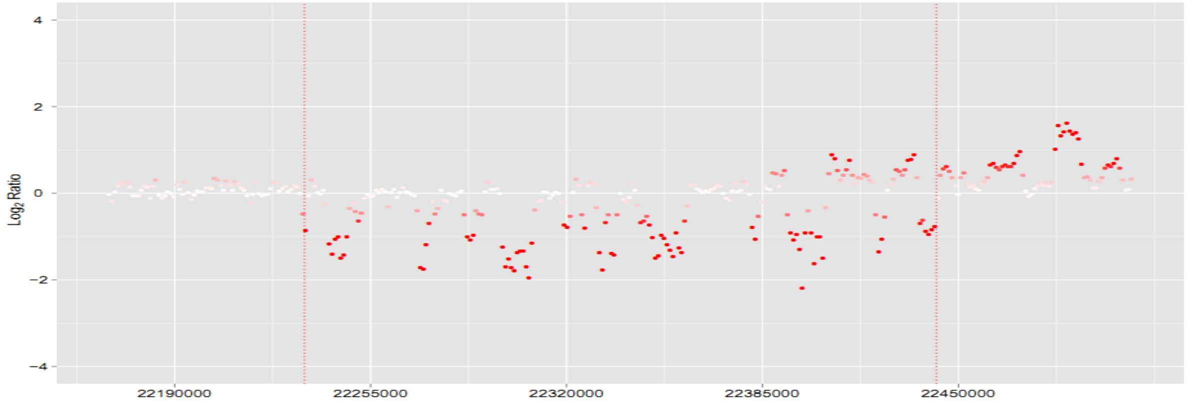

Complete Genomics This Work

NA07357_CEUI_R1b1b2

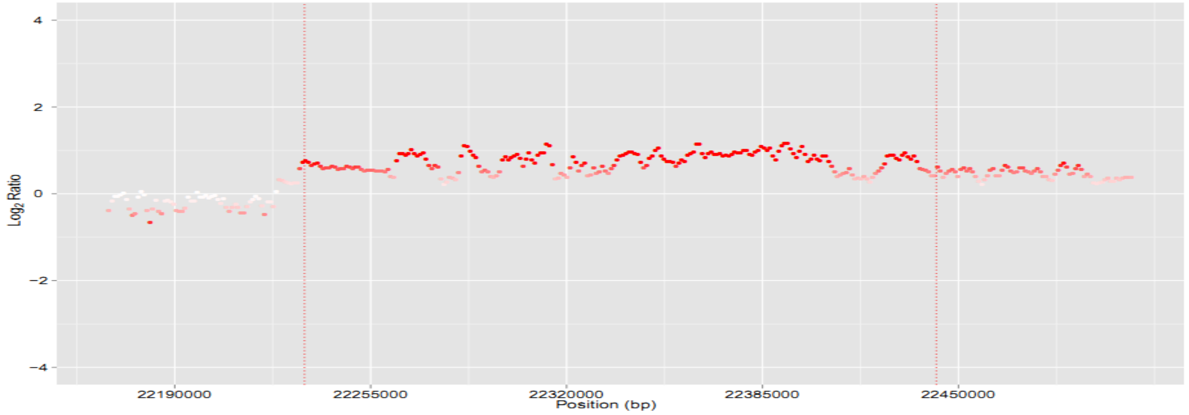

Segmental Duplications

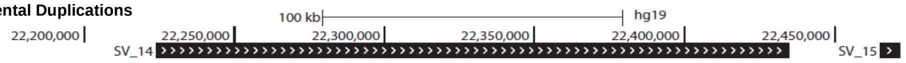

Region SV 15
DYZ19
Evidence: P1T + CGR + Ph1R
From 22,464,987 To 22,471,737
Length: 6,750 bp
Present in 14 samples

Pilot 1 This Work NA18944_JPT_D

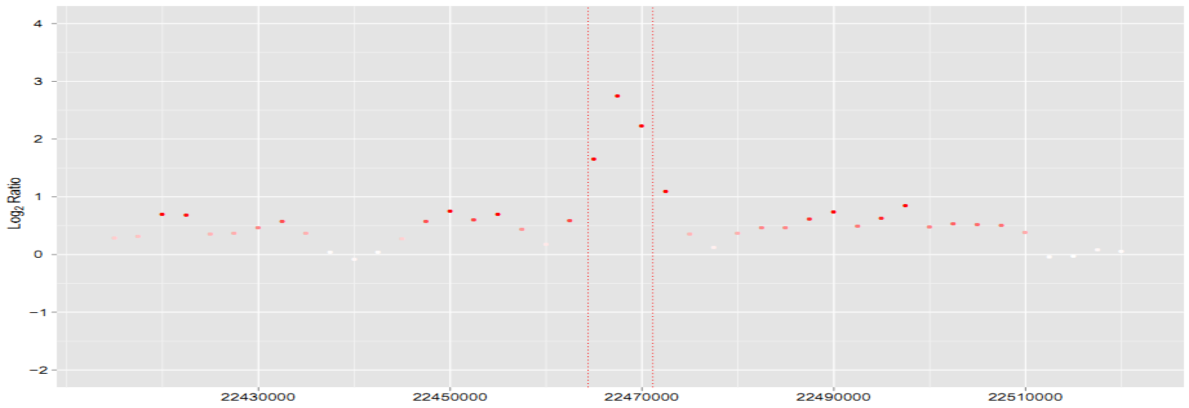

Pilot 1 This Work - Haplogroup Analysis Haplogroup_C

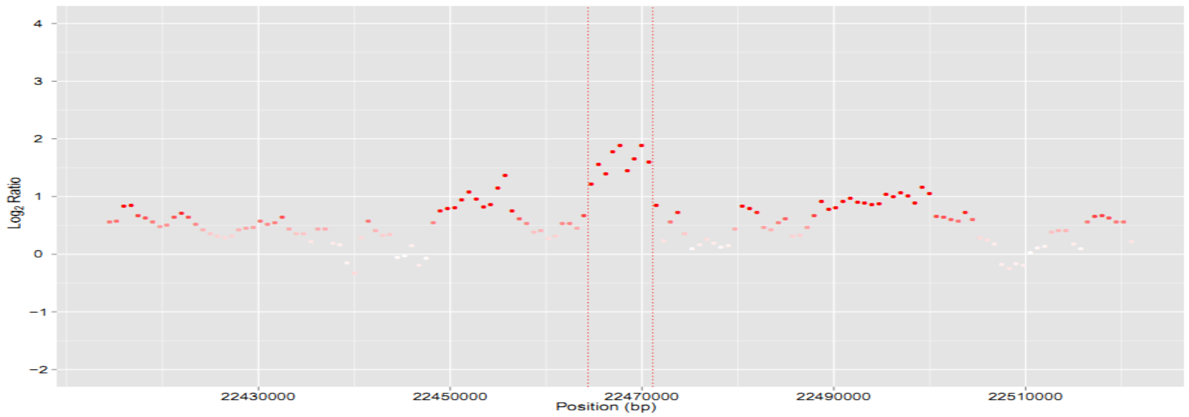

Segmental Duplications

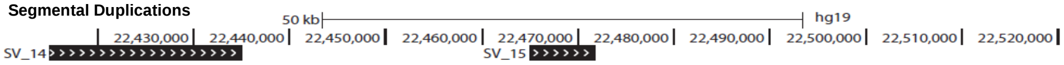

Region SV 16
gr/gr deletion
Evidence: P1T + CGT + CGR + OCT + L + PCR
From 24,875,619 To 26,526,445
Length: 1,650,826 bp
Present in 12 samples

Pilot 1 This Work NA18504_YRI_E1b1a

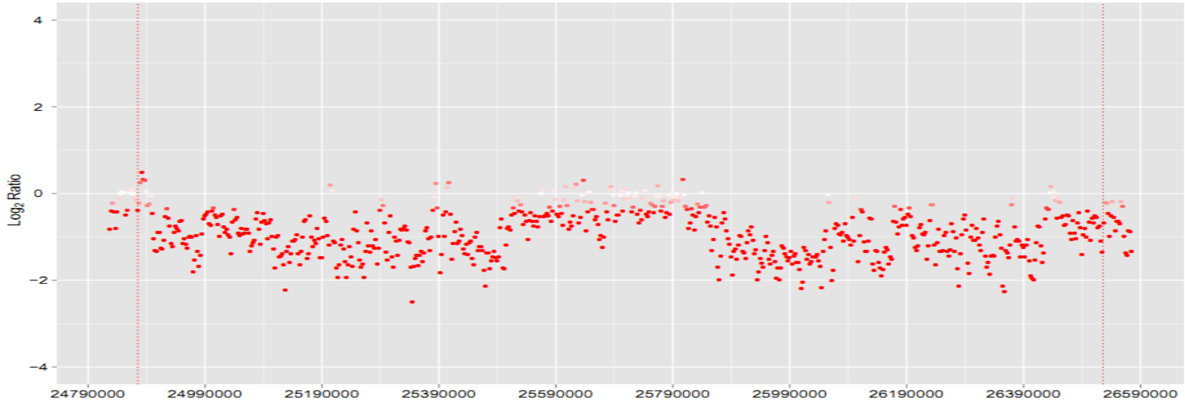

Pilot 1 This Work - Haplogroup Analysis Haplogroup_D

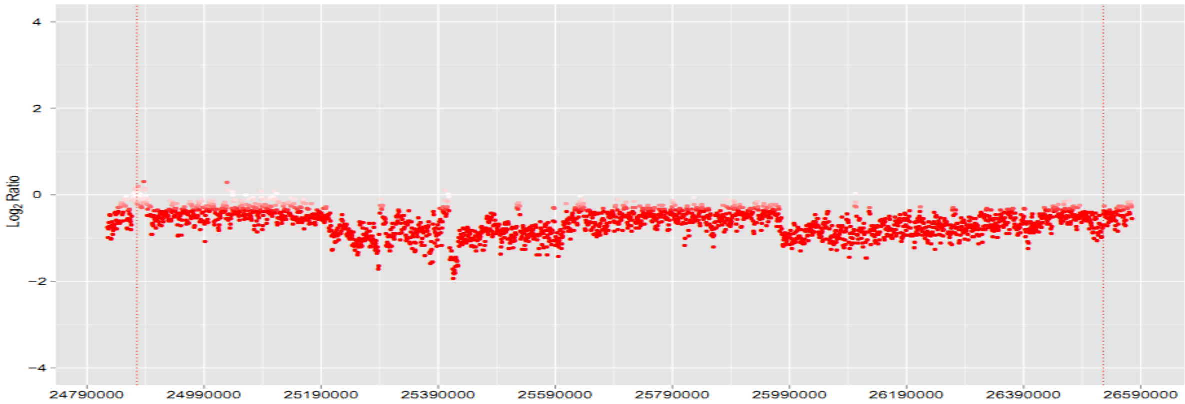

Complete Genomics This Work NA18504_YRI_E1b1a

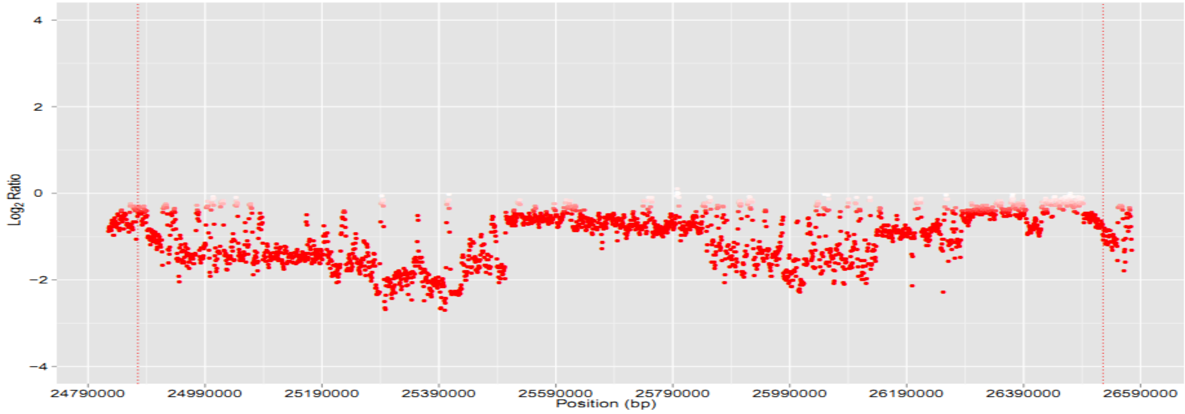

Segmental Duplications

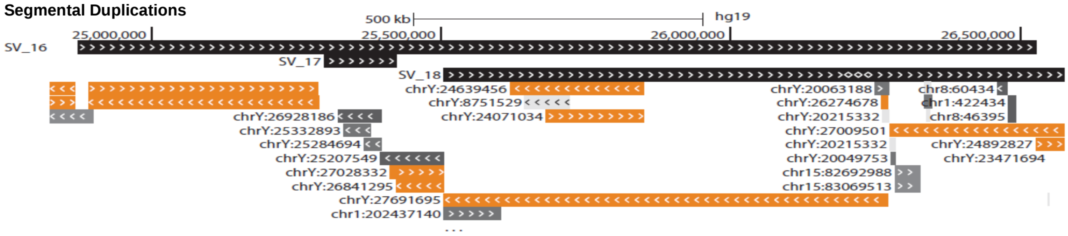

OMNI Chip This Work - Event

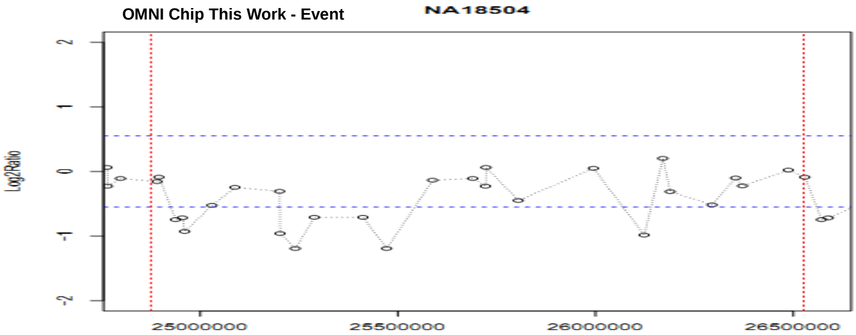

OMNI Chip This Work - No Event

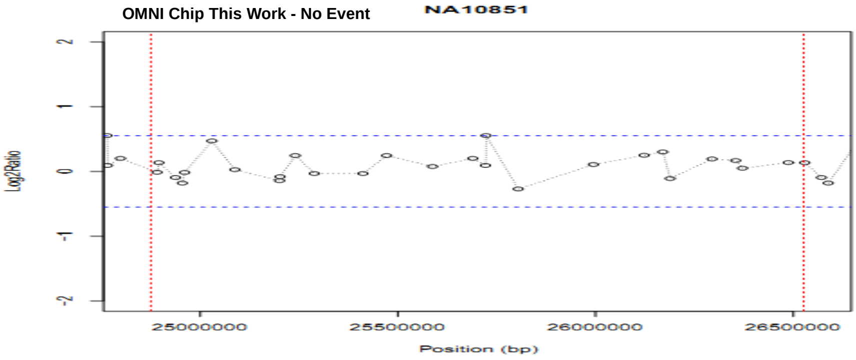

PCR

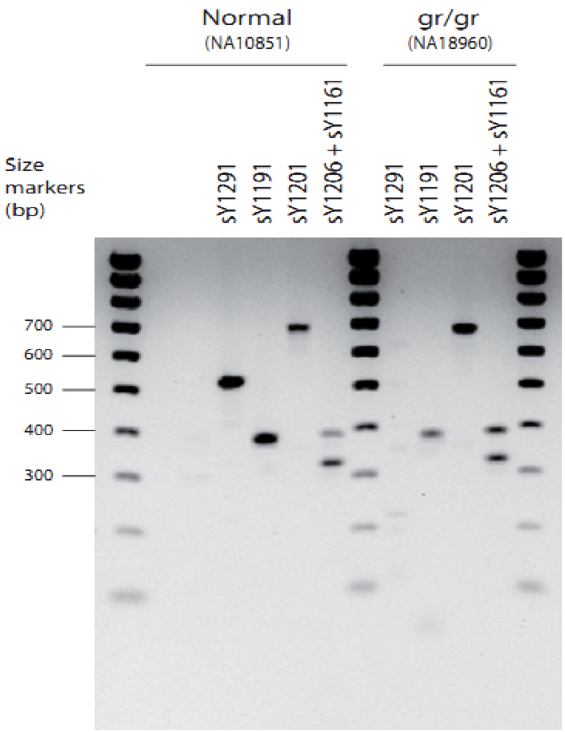
Normal
(NA10851)
gr/gr
(NA18960)
Size markers (bp)
sY1291
sY1191
sY1201
sY1206 + sY1161
sY1291
sY1191
sY1201
sY1206 + sY1161
700
600
500
400
300

Region SV 17
DAZ 1/2
Evidence: P1T + CGT + CGR + L
From 25,299,362 To 25,424,362
Length: 125,000 bp
Present in 19 samples

Pilot 1 This Work NA12043_CEU_R1

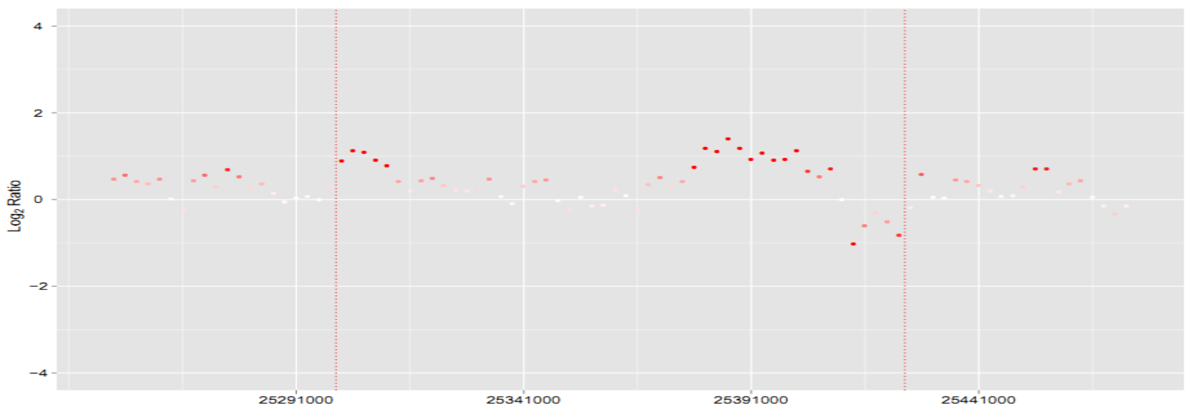

Pilot 1 This Work - Haplogroup Analysis Haplogroup_R1

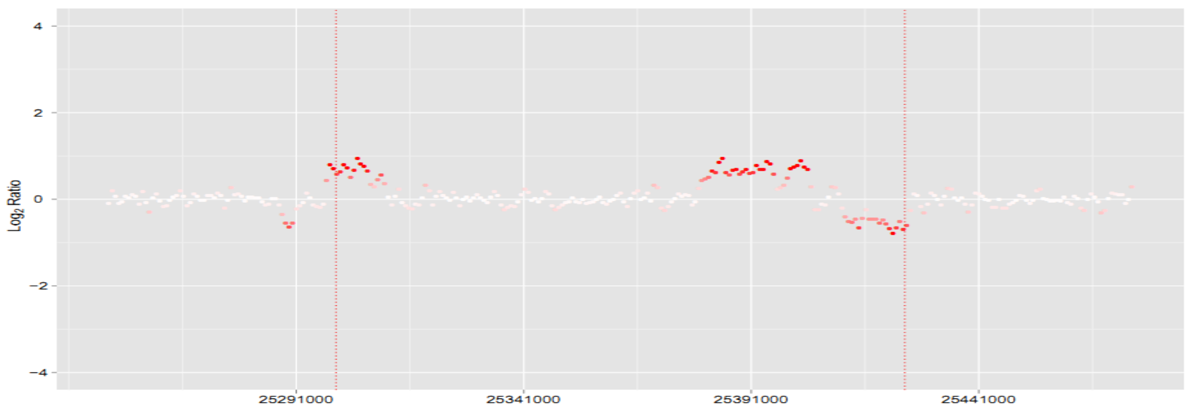

Complete Genomics This Work NA10851_CEU_R1

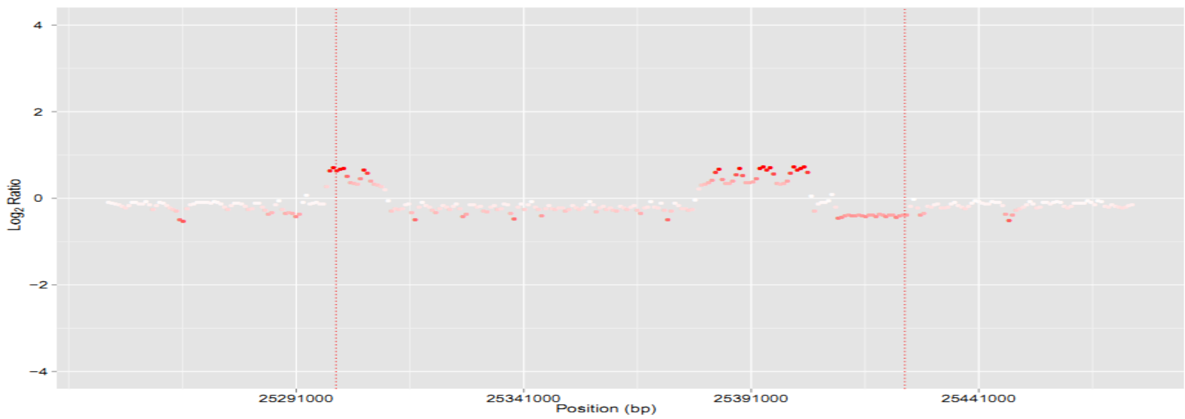

Segmental Duplications

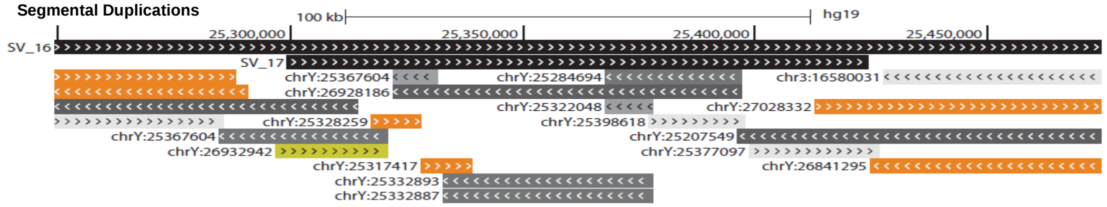

Region SV 18
b2/b3 (g1/g3) deletion
Evidence: L + PCR
From 25,505,069 To 27,435,593
Length: 1,930,524 bp
Present in 3 samples

PCR

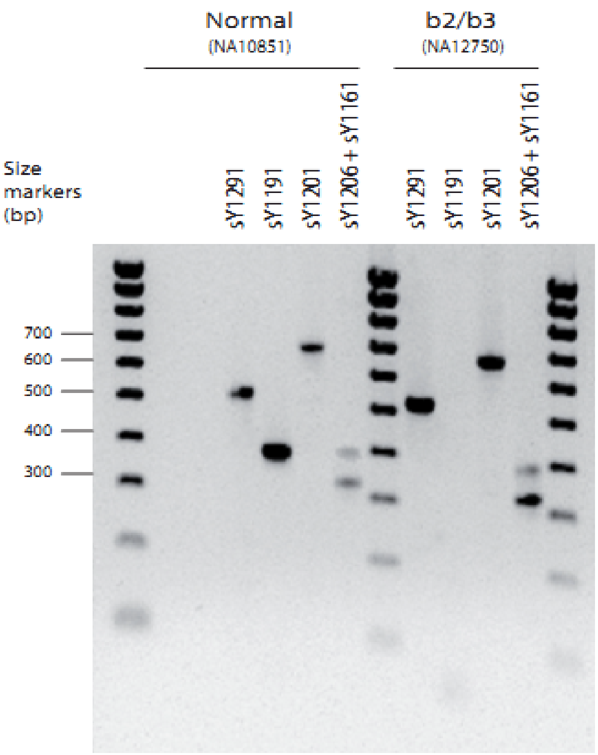

Segmental Duplications

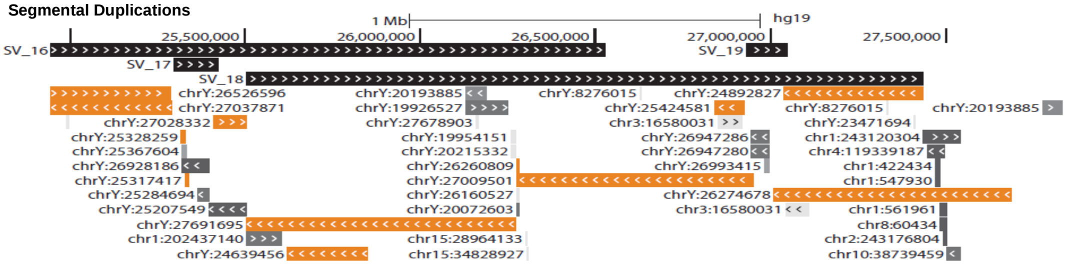

Region SV 19
DAZ 3/4
Evidence: P1T + CGT + CGR + L
From 26,929,362 To 27,046,862
Length: 117,500 bp
Present in 19 samples

Pilot 1 This Work

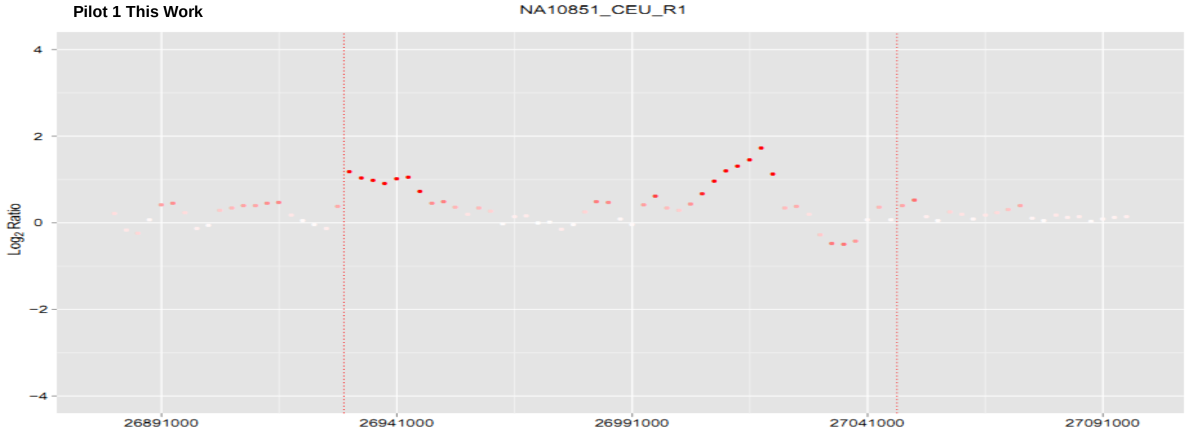

Pilot 1 This Work - Haplogroup Analysis

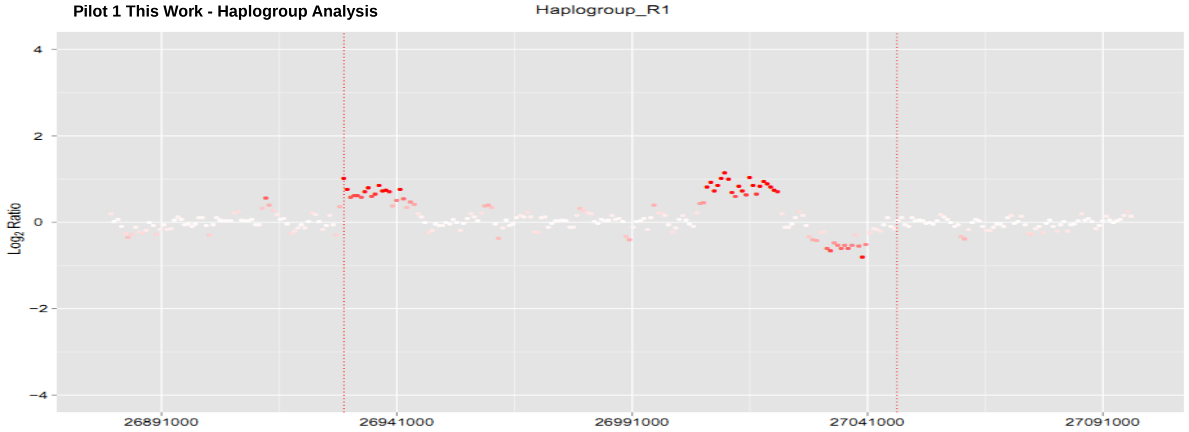

Complete Genomics This Work

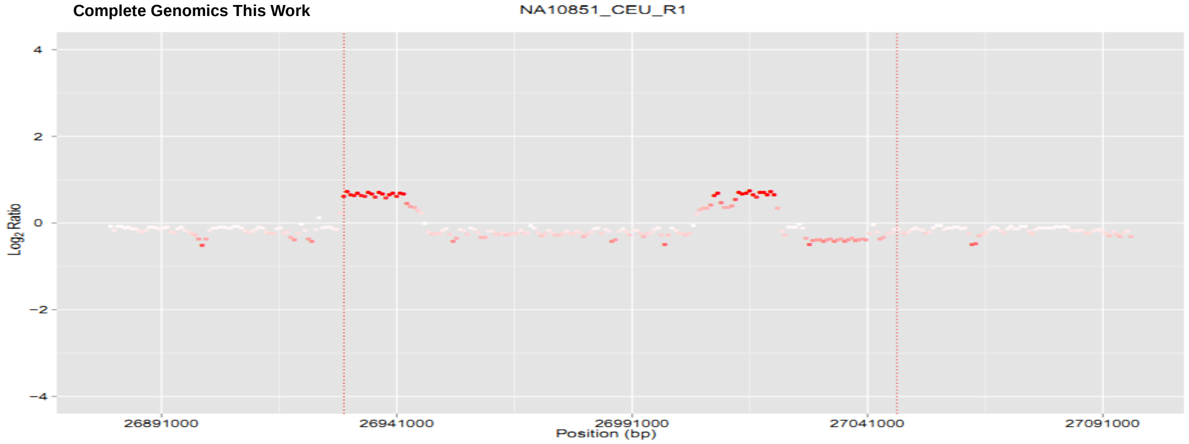

Segmental Duplications

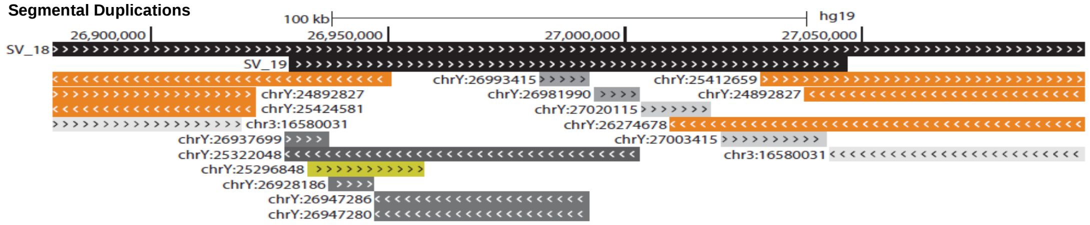

Supplement: Supplementary material [file CroatMedJ_56_s005.pdf]
